# Balancing Selection Maintains a Form of *ERAP2* that Undergoes Nonsense-Mediated Decay and Affects Antigen Presentation

**DOI:** 10.1371/journal.pgen.1001157

**Published:** 2010-10-14

**Authors:** Aida M. Andrés, Megan Y. Dennis, Warren W. Kretzschmar, Jennifer L. Cannons, Shih-Queen Lee-Lin, Belen Hurle, NISC Comparative Sequencing Program, Pamela L. Schwartzberg, Scott H. Williamson, Carlos D. Bustamante, Rasmus Nielsen, Andrew G. Clark, Eric D. Green

**Affiliations:** 1 Genome Technology Branch, National Human Genome Research Institute, National Institutes of Health, Bethesda, Maryland, United States of America; 2 Genetic Disease Research Branch, National Human Genome Research Institute, National Institutes of Health, Bethesda, Maryland, United States of America; 3 NIH Intramural Sequencing Center, National Human Genome Research Institute, National Institutes of Health, Bethesda, Maryland, United States of America; 4 Department of Biological Statistics and Computational Biology, Cornell University, Ithaca, New York, United States of America; 5 Department of Integrative Biology, University of California, Berkeley, Berkeley, California, United States of America; 6 Department of Molecular Biology and Genetics, Cornell University, Ithaca, New York, United States of America; National Institute of Genetics, Japan

## Abstract

A remarkable characteristic of the human major histocompatibility complex (MHC) is its extreme genetic diversity, which is maintained by balancing selection. In fact, the MHC complex remains one of the best-known examples of natural selection in humans, with well-established genetic signatures and biological mechanisms for the action of selection. Here, we present genetic and functional evidence that another gene with a fundamental role in MHC class I presentation, endoplasmic reticulum aminopeptidase 2 (*ERAP2*), has also evolved under balancing selection and contains a variant that affects antigen presentation. Specifically, genetic analyses of six human populations revealed strong and consistent signatures of balancing selection affecting *ERAP2*. This selection maintains two highly differentiated haplotypes (Haplotype A and Haplotype B), with frequencies 0.44 and 0.56, respectively. We found that *ERAP2* expressed from Haplotype B undergoes differential splicing and encodes a truncated protein, leading to nonsense-mediated decay of the mRNA. To investigate the consequences of ERAP2 deficiency on MHC presentation, we correlated surface MHC class I expression with *ERAP2* genotypes in primary lymphocytes. Haplotype B homozygotes had lower levels of MHC class I expressed on the surface of B cells, suggesting that naturally occurring ERAP2 deficiency affects MHC presentation and immune response. Interestingly, an *ERAP2* paralog, endoplasmic reticulum aminopeptidase 1 (*ERAP1*), also shows genetic signatures of balancing selection. Together, our findings link the genetic signatures of selection with an effect on splicing and a cellular phenotype. Although the precise selective pressure that maintains polymorphism is unknown, the demonstrated differences between the *ERAP2* splice forms provide important insights into the potential mechanism for the action of selection.

## Author Summary

It has long been known that the extremely high levels of genetic diversity present in the major histocompatibility locus (MHC) are due to balancing selection, a type of natural selection that maintains advantageous genetic diversity in populations. The MHC encodes for molecules required for a type of antigen presentation that mediates detection of infected and cancerous cells by the immune system; the genetic diversity of the MHC thus ensures an adequate response to the wide variety of pathogens that humans encounter. Here, we show that other genes involved in the same antigen-presentation pathway are also subject to balancing selection in humans. Specifically, we show that balancing selection acts to maintain two forms of the endoplasmic reticulum aminopeptidase 2 gene (*ERAP2*), which encodes a protein also involved in antigen presentation. Although the two *ERAP2* forms are present in a similar frequency (close to 0.5), they are associated with differences with respect to the levels of MHC molecules on the cell surface of immune cells. In summary, our findings show that natural selection maintains variants of *ERAP2* that affect immune surveillance; they also establish *ERAP2* as one of the few examples of balancing selection in humans where the selected variant, its functional consequences, and its influence in interpersonal diversity are known.

## Introduction

Balancing selection maintains advantageous genetic diversity in populations. Unlike positive and purifying selection, which favor fixation of the fittest allele, balancing selection results in enhanced genetic and phenotypic variability in populations. Diversity can be maintained by overdominance (the higher fitness of heterozygotes), frequency-dependent selection (when an allele's effect on fitness varies with its frequency), fluctuating selection (selection that changes in time or space), or pleiotropy (selection on a variant that affects multiple traits). Over time, all of these processes leave the characteristic genetic footprint of balancing selection: an excess of polymorphism due to the long-term maintenance of selected alleles, and an enrichment of variants with a frequency close to the frequency equilibrium (for example, an enrichment in variants at intermediate frequency if the optimal frequency of the selected variant is 0.5).

These, and related signatures allow the identification of candidate targets of balancing selection [1]–[3]. However, discerning the biological processes underlying balancing selection remains a challenge, even for loci with striking genetic signatures. As a result, there are few well-characterized examples of balancing selection in humans, with both clear genetic signatures and a known biological mechanism for the action of selection. One prominent exception is the major histocompatibility complex (*MHC*) class I locus, arguably the best-established target of natural selection in vertebrates [4]–[8]. The *MHC* class I locus is extremely polymorphic (over 3000 alleles have been described in humans; see ebi.ac.uk/imgt/hla/stats.html) and some of its ancestral polymorphism has been maintained for millions of years in several extant species (i.e., trans-species polymorphism) [9]. Such extreme variability ensures MHC presentation of highly diverse antigenic peptides and, in turn, allows the detection of many different pathogens, improving the effectiveness of the immune system.

Interestingly, another component involved in MHC function, the natural killer-cell proteins that recognize MHC-peptide complexes (killer-cell immunoglobulin-like receptors, KIR), show signatures of balancing selection and coevolution with *MHC* class I [10]–[12]. The crucial role that MHC-mediated antigen presentation plays on individual survival explains the influence that balancing selection has on the evolution of *MHC* and *KIR*. In addition, we recently identified another key element of the MHC class I antigen-presentation process as a candidate target of balancing selection: endoplasmic reticulum aminopeptidase 2 (*ERAP2*) [3].

The MHC class I-dependent antigen presentation pathway starts with the degradation of intracellular proteins by cytoplasmic proteases. Some of the resulting short peptides are translocated into the endoplasmic reticulum for the final trimming of their N-terminal residues by ERAP2 and its paralog, ERAP1. The two proteins show different peptide specificity, and they act in a concerted fashion to generate peptides of the appropriate length and sequence for MHC class I binding and presentation. Once the MHC molecule and peptide are coupled, the complex is translocated to the cell surface, where presentation takes place. By performing the final trimming steps that ensure the presence of optimal MHC class I ligands, ERAP1 and ERAP2 play a key role in MHC antigen presentation (reviewed in [13]–[19]).

In addition to a role in peptide MHC class I presentation, ERAP1 and ERAP2 contribute to a number of other biological processes. Both genes are regulated by interferon γ IFN- γ and are involved in immune activation and inflammation [20]. They may also regulate angiogenesis and blood pressure [21], [22] through the trimming of angiotensin II and angiotensin III, respectively [23], [24]. *ERAP1* and *ERAP2* are down-regulated in some tumors, suggesting a role in the detection of transformed cells by immune surveillance [25], [26]. *ERAP1* genetic variants are associated with ankylosing spondylitis [27]–[30], and cervical carcinoma [31]–[33]. Meanwhile, *ERAP2* variants and expression levels have been associated with pre-eclampsia [34], [35], a dangerous hypertensive complication of pregnancy with both immunological and inflammatory components. Haroon and Inman [36] provide a more comprehensive review of the pathogenic potential of *ERAP1* and *ERAP2*. Of note, *ERAP2* has not been studied as extensively as *ERAP1* because of its absence in rodent (e.g., mouse, rat, and guinea pig) genomes, although its phylogeny reveals that it was present in the primate-rodent common ancestor (genome.ucsc.edu).

Our earlier genomic study revealed increased polymorphism and the genetic signatures of balancing selection in *ERAP2* in African-Americans and European-Americans [3]. Based on these data, we hypothesized that advantageous genetic diversity might enhance not only antigen presentation and recognition (e.g., *MHC* and *KIR*), but also earlier steps of the MHC antigen presentation pathway. Here, we present evidence to support this hypothesis. Specifically, we show that *ERAP2* has distinct signatures of balancing selection in geographically diverse human groups, and that, interestingly, *ERAP1* shows similar signatures of selection. Furthermore, we provide bioinformatic, molecular, cellular, and immunological evidence that identifies an *ERAP2* putatively selected variant, establishes its effect on protein function, and demonstrates a downstream impact on MHC class I presentation.

## Results

### *ERAP2* evolution

*ERAP2* is a 19-exon gene located on human chromosome 5q15, residing between *ERAP1* (in the opposite orientation and likely sharing regulatory elements) and leucyl-cystinyl aminopeptidase (*LNPEP*); see Figure S1. We sequenced the complete protein-coding sequence (cds) and adjacent non-coding regions of *ERAP2* in 180 individuals from 6 human populations: Luhya, Yoruba, Palestinian, Gujarati, Han, and Toscani. From these data, we identified 22 coding single-nucleotide polymorphisms (SNPs) and 57 non-coding SNPs. As a proxy for neutrality, we also sequenced 47 neutral genomic segments (i.e., control regions, see Materials and Methods for details), identifying 287 SNPs within our sample set.

**Figure 1:**
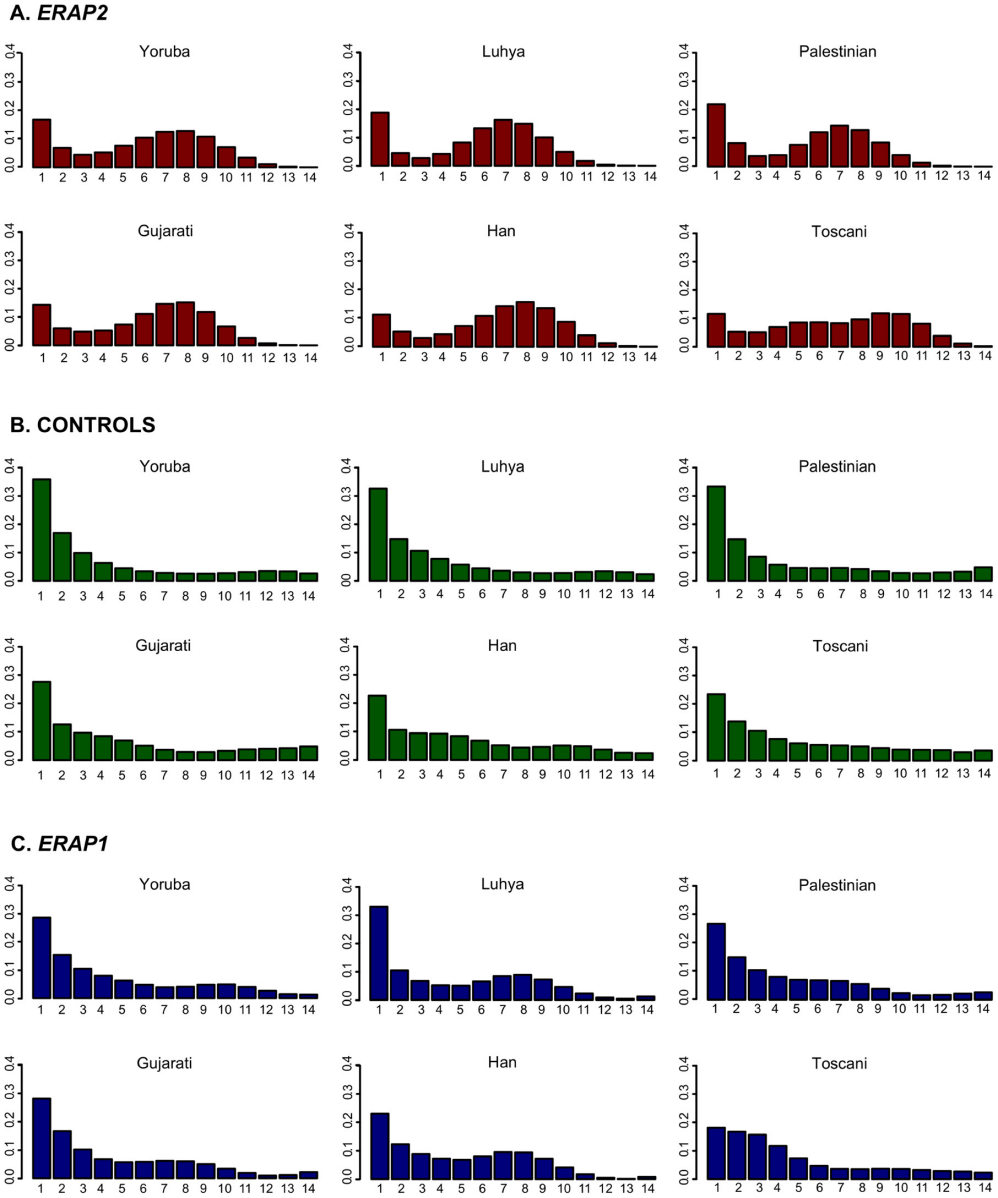
Allele site-frequency spectrum (SFS) of *ERAP2*, control regions, and *ERAP1* in each population. The X-axis reflects the absolute frequency of the derived allele, while the Y-axis reflects the frequency of that allele frequency bin in the generated data set. To account for missing data, the frequencies were projected to a sample size of 15 chromosomes. See the SFS of only coding SNPs in Figure S2.

Figure 1A and 1B depicts the distribution of allele frequencies (i.e., the allele site frequency spectrum, SFS) for *ERAP2* and the control regions, respectively. With the control regions, the SFS shows a distinct skew towards low-frequency variants, as is typically seen in human datasets [37]. In contrast, with *ERAP2*, there is a marked enrichment in intermediate-frequency variants. This excess of intermediate-frequency alleles is significant in all populations based on both the MWUhigh test [3], [37] and Tajima's D analysis [38] (Table 1). Analyses of only coding SNPs reveal the same trend (Figure S2 and Table S1). Overall, *ERAP2* shows strong and consistent signatures of balancing selection maintaining intermediate-frequency alleles.

**Table 1:** Neutrality tests.

Population	All SNPs				Coding SNPs				Coding
	S	TajD	p(TajD)	p(MWU)	S	TajD	p(TajD)	p(MWU)	p(HKA)
***ERAP2***									
Yoruba	45	2.05	0	0	10	1.43	0.004	0.017	0.525
Luhya	51	1.44	0.000	0.000	11	0.95	0.026	0.145	0.400
Palestinian	55	1.34	0.004	0.001	13	1.05	0.094	0.028	0.019
Gujarati	45	1.99	0	0	12	1.05	0.105	0.068	0.033
Han	38	2.68	0	0	9	1.95	0.008	0.001	0.150
Toscani	40	2.30	0	0	11	1.17	0.078	0.085	0.067
***ERAP1***									
Yoruba	52	0.19	0.032	0.048	20	0.10	0.185	0.242	0.016
Luhya	55	−0.06	0.113	0.139	19	0.16	0.158	0.201	0.023
Palestinian	58	0.38	0.196	0.038	22	0.08	0.435	0.311	0
Gujarati	54	0.44	0.173	0.082	22	0.18	0.382	0.327	0.000
Han	41	0.91	0.027	0.010	18	0.55	0.202	0.131	0.000
Toscani	49	1.07	0.012	0.007	17	1.16	0.057	0.037	0.002

The number of SNPs (**S**) and results for the three neutrality tests performed for *ERAP2* and *ERAP1* using data generated from the six populations are indicated [**TajD**: Tajima's D; **p(TajD)**: *P*-value for Tajima's D test; **p(MWU)**: *P*-value for MWUhigh test; **p(HKA)**: *P*-value for HKA test]. HKA was performed only for the coding regions of the genes. The complete matrix with summary statistics is presented in Table S1.

Our analyses of *ERAP2* revealed 22 coding SNPs and 10 coding fixed differences with chimpanzee: 2.2 coding SNPs per fixed difference. This represents a 2.7-fold enrichment compared with the control regions, which have 0.82 SNPs per fixed difference (287 SNPs and 352 fixed differences). The excess of polymorphism is significant in two populations (Palestinian and Gujarati) and marginally non-significant in the Toscani group (HKA test [39], Table 1), but fails to reach significance in the other populations (likely due to the limited power of the short coding regions). Consistent with a relatively long-term influence of selection, *ERAP2* does not show the characteristic long-range linkage disequilibrium (LD) of very recent balancing selection (Figure S3 and Text S1); the estimated coalescent time of the locus is 1.44 Mya (standard deviation: 550,000 years).

**Figure 2:**
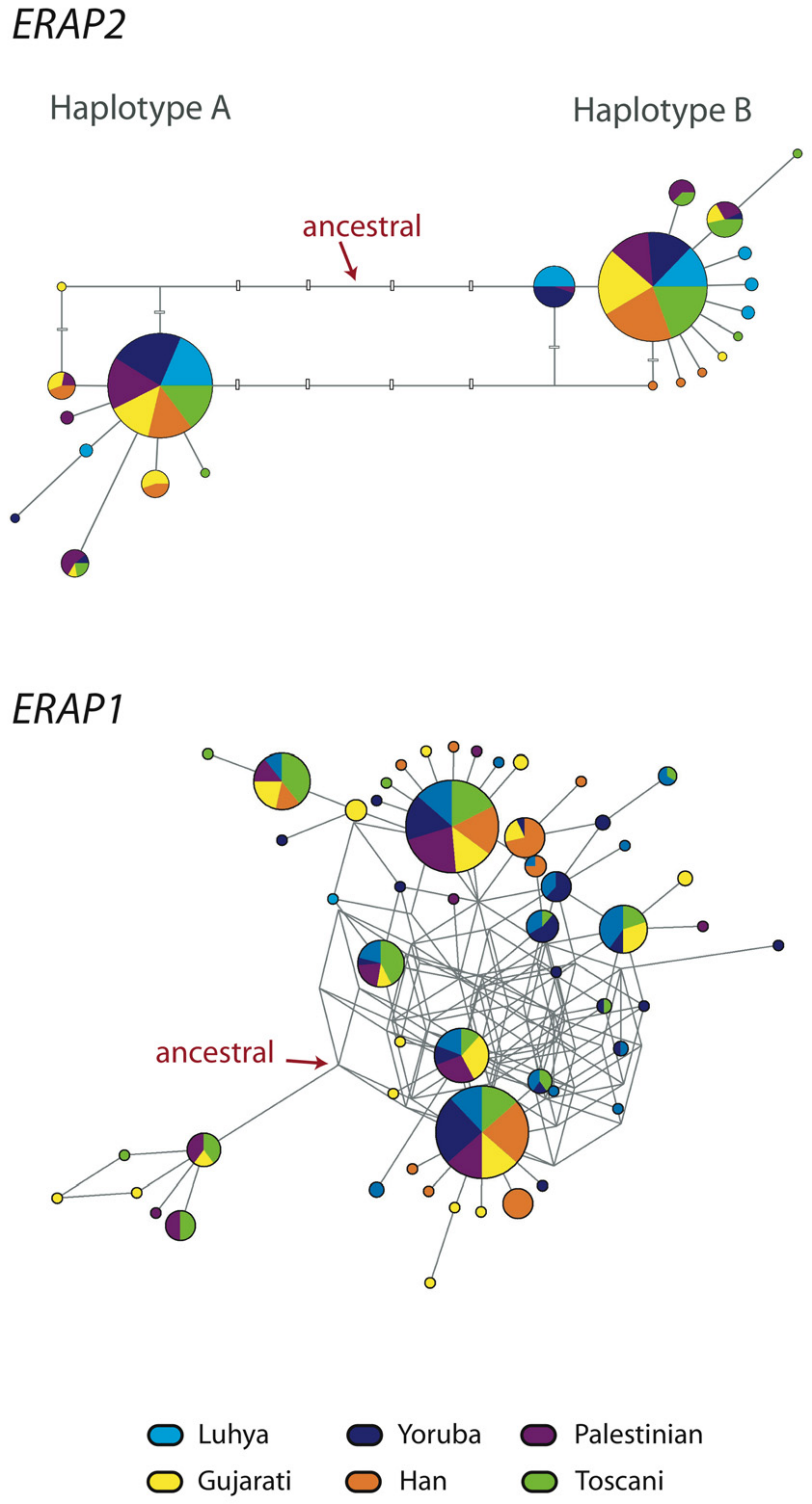
Haplotype network of *ERAP2* and *ERAP1*. Circles represent haplotypes, with the areas proportional to the frequency of the haplotype (color-coded by population). The lines connecting the haplotypes have a length proportional to the number of mutations that differentiate the two haplotypes. Reticulations reflect recombinations or recurrent mutations. The ancestral state was inferred using the chimpanzee sequence data. For *ERAP2*, the four coding diagnostic SNPs are shown as white boxes; one nearly diagnostic SNP, which appears four times in the network due to the reticulations, is marked as thinner horizontal boxes. The *ERAP2* haplotype network that includes all SNPs (coding and non-coding) is shown in Figure S5, and the *ERAP2* haplotype network that includes the chimpanzee sequence is shown in Figure S6.

The haplotype network of *ERAP2* is highly structured, with two differentiated clades or haplogroups: ‘Haplotype A’ and ‘Haplotype B’ (Figure 2). The two haplotypes are differentiated by numerous SNPs, including four coding SNPs and a large number of non-coding SNPs (not depicted). We refer to these SNPs as ‘diagnostic SNPs.’ Each haplotype has a frequency around 0.5 in all populations (Figure 2), with the ancestral state set between the two haplotypes. The similar distribution of variants in the two haplogroups and their similar patterns of long-range LD (see above), points to a similar age for each. Taken together, the signatures of selection and the maintenance of two haplogroups at similar frequencies suggest a functional difference between Haplotype A and Haplotype B.

### Effects of *ERAP2* variants on mRNA splicing

We identified four coding diagnostic SNPs that differentiate the coding sequence of Haplotype A and Haplotype B. Only one of these reflects a non-synonymous variant, resulting in a conservative change unlikely to influence protein function (K392N, a basic polar residue to a neutral polar). Nevertheless, several studies have previously identified associations between SNPs in this genomic region and changes in *ERAP2* expression and splicing [40]–[43]. In addition, a recent study identified an intronic variant that is associated with differential splicing of *ERAP2* [44]. These studies suggest that *ERAP2* variants can alter splicing, raising the possibility of differences in the splicing of *ERAP2* mRNA expressed from Haplotype A versus Haplotype B.

**Figure 3:**
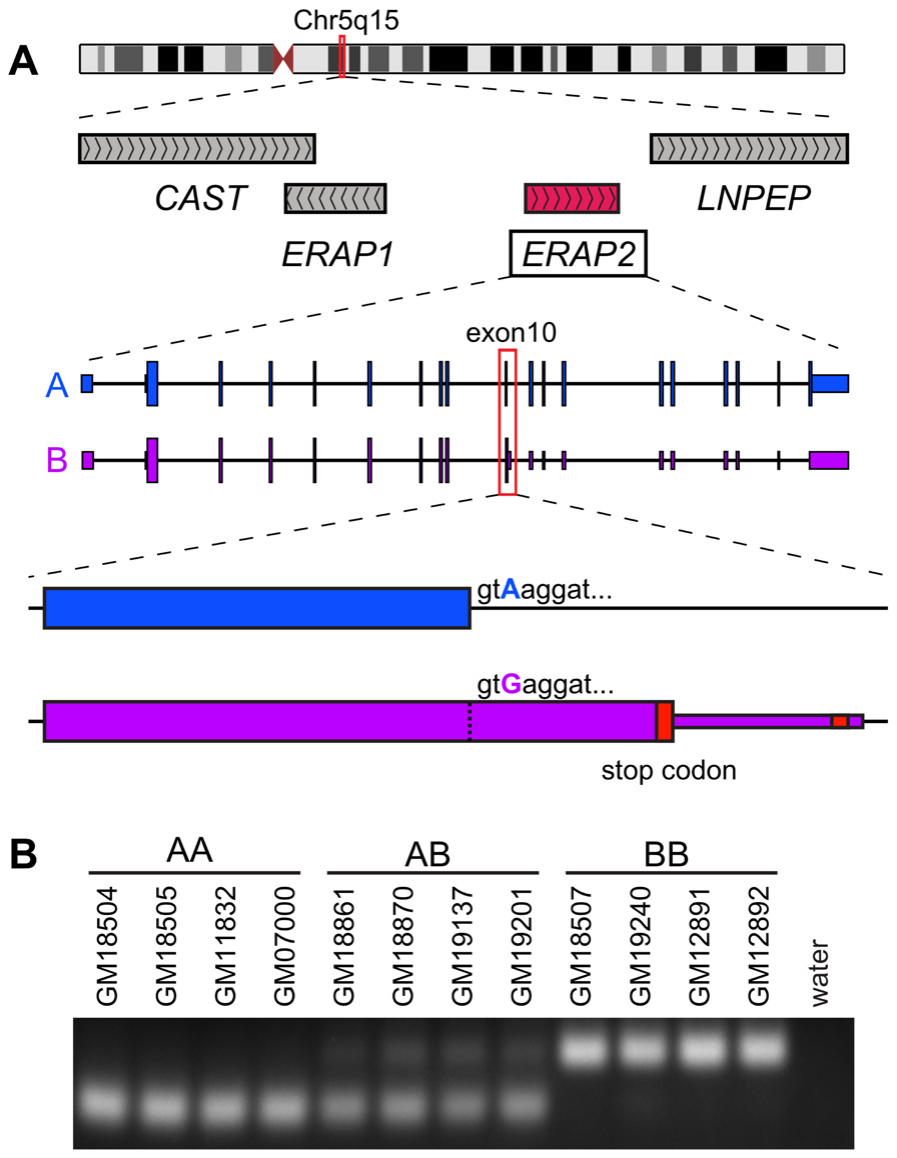
Haplotype-specific splicing of *ERAP2*. A, The genomic organization of the human chromosome 5q15 region containing *ERAP1* and *ERAP2* is included at the top. The two haplotype-specific *ERAP2* spliced forms are shown for Haplotype A (in blue) and Haplotype B (in purple). The different alleles of rs2248374 are shown as a blue or purple base position, respectively. The red boxes represent the premature stop codons in the Haplotype B mRNA. B, PCR amplification of cDNA across the exon 10 splice junction (see Materials and Methods) from the indicated 16 LCLs, with the haplotype status of each cell indicated as homozygote (AA or BB) or heterozygote (AB). A negative control PCR, with no DNA template, was also performed (water).

To explore this hypothesis, we sequenced the complete *ERAP2* cDNA isolated from EBV-transformed lymphoblastoid cell lines (LCLs) derived from two HapMap individuals: one homozygous for Haplotype A (AA-homozygote) and one homozygous for Haplotype B (BB-homozygote). We used LCLs because *ERAP2* is highly expressed in lymphocytes [45] and this cell type is particularly relevant for studies of MHC class I presentation. One identified splicing form, which contains an extended exon 10 with 56 extra nucleotides (AY028805.1 and AB163917.1 [20]), was observed only in Haplotype-B mRNAs (Figure 3A). To confirm that this splice form is indeed specific to Haplotype B, we used PCR to isolate from cDNA the region across the exon 10 and exon 11 splice junction in 12 HapMap LCLs with varied genotypes (Figure 3B). The exon 10 ‘extension’ was detected in all 4 BB-homozygotes but none of the 4 AA-homozygotes; both splice forms were detected in AB-heterozygotes. Therefore, Haplotype A-expressed *ERAP2* is consistently spliced to contain the standard exon 10, while Haplotype B-expressed *ERAP2* is spliced to contain the extended version of exon 10. These results are consistent with an *in silico* analysis of all publicly available *ERAP2* mRNAs and ESTs (Text S1). We conclude that the haplotype-specific splicing of *ERAP2* must be driven by a diagnostic SNP.

Extension of exon 10 occurs when the standard splice site (position 69 of exon 10) is skipped in favor of a downstream cryptic splice site at position 56 of intron 10. Only one diagnostic SNP resides in the proximity of exon 10: rs2248374, which lies within the 5′ canonical splice site (Figure 3A). Haplotype A contains the rs2248374-A allele, while Haplotype B contains the rs2248374-G allele. *In silico* prediction of optimal splicing (GeneID [46]) with the rs2248374-A allele yields the Haplotype A splice form, while prediction with the rs2248374-G allele yields the Haplotype B splice form (Text S1). According to MaxEnt, a maximum entropy computational analysis of splice sites [47], and as shown by Coulombe-Huntington et al. [44], this is due to rs2248374 reducing the signal strength of the exon 10 donor splice site from 9.33 (for the A allele) to 7.61 (for the G allele). Coulombe-Huntington et al. [44] studied 78 candidate loci of allele-specific splicing, and experimentally confirmed 6 of them, including rs2248374 and *ERAP2* exon 10. Together, these results show that the difference in *ERAP2* splicing between Haplotypes A and B is due to rs2248374, whose A and G alleles increase and reduce the strength of the splice site, respectively.

### Effects of *ERAP2* variants on mRNA processing and translation

**Figure 4:**
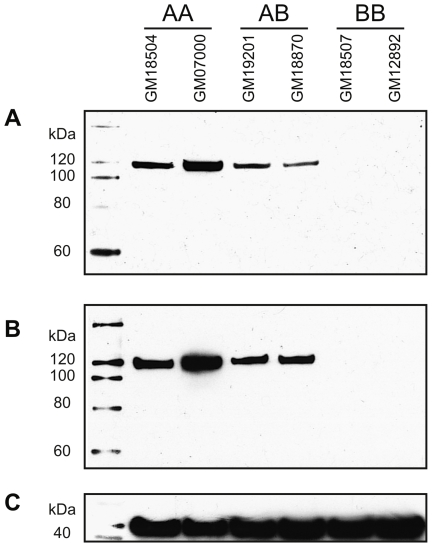
Immunoblot analyses of ERAP2 using LCL protein extract. Two LCLs of each *ERAP2* genotype (AA, AB, and BB) were tested for protein using primary antibodies specific to: A, ERAP2 (goat polyclonal); B, ERAP2 (mouse polyclonal); and C, ß-actin (see Materials and Methods).

The *ERAP2* mRNA derived from Haplotype A encodes the canonical (full-length) ERAP2 protein consisting of 960 amino acids. In contrast, translation of the *ERAP2* mRNA derived from Haplotype B would be predicted to produce a truncated protein of 534 amino acids, since the exon 10 extension contains two TAG stop codons (Figure 3A). This second mRNA form was first reported in an early characterization of the gene [24]. We sought to detect the truncated form of ERAP2 by western blot analysis of protein extracted from LCLs using two antibodies that should detect both truncated and full-length forms of the protein. This analysis revealed that AA-homozygote cells produce only full-length ERAP2 (120 kDa), while BB-homozygote cells produce no detectable ERAP2 protein (Figure 4). Additionally, AB-heterozygotes only produce full-length ERAP2, in seemingly smaller quantities compared to AA-homozygotes (the intensity of the full-length ERAP2 band in AB-heterozygotes is 35% and 50% that of AA-homozygotes for the two antibodies, respectively). Therefore, only the full-length ERAP2 protein is detectable in LCLs, and only in AA-homozygotes and AB-heterozygotes.

We did detect an extremely faint band in BB-homozygotes, of the size of the full-length ERAP2 protein, when the western was run with mouse 3F5 antibody [48] (Figure S4). This band could be due to unspecific binding of the mouse mAb 3F5 antibody, since unspecific bands were observed in that experiment (Figure S4); however, if it corresponds to ERAP2 it likely derives from the very limited amount of *ERAP2* Haplotype B that is spliced to contain the canonical exon 10 (Figure 3B). This small amount of protein likely has no or very little biological relevance, particularly when compared with the high levels observed in AA-homozygotes and AB-heterozygotes. In any case, note that truncated ERAP2 protein (60 kDa) could not be detected in this experiment (Figure S4).

**Figure 5:**
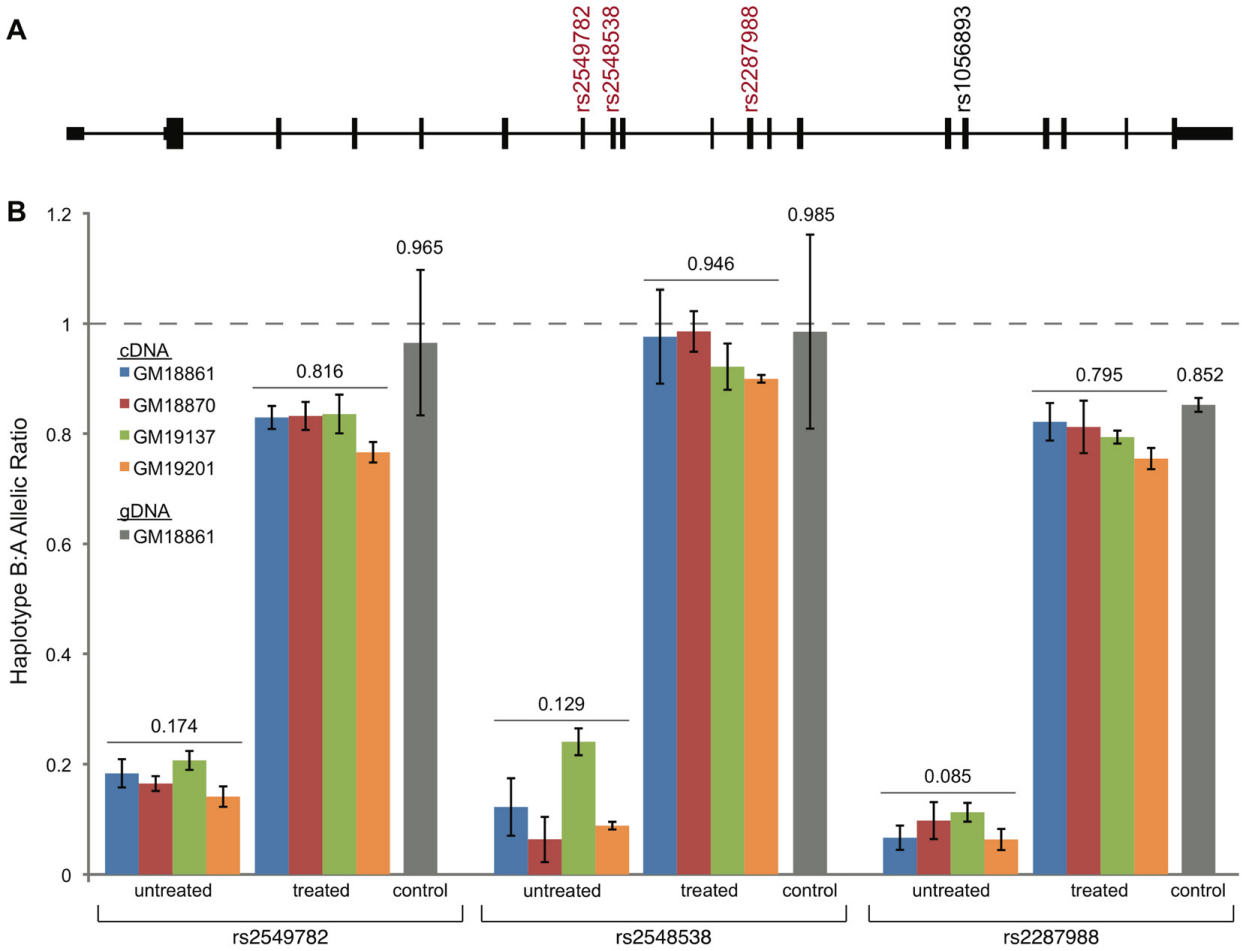
Quantification of allele-specific *ERAP2* mRNA levels in LCLs. A, Locations of the four coding diagnostic SNPs across *ERAP2* are shown, of which three (in red) were used to test for allele-specific expression. B, The allelic ratio of Haplotype B to Haplotype A *ERAP2* cDNA levels, which was measured using these three coding diagnostic SNPs in the indicated heterozygote LCLs treated/untreated with emetine (NMD blocked), are depicted with colored bars. The control represents the allelic ratio measured with genomic DNA (gDNA), expected to be 1.0. The average allelic ratio across all cell lines tested (for a given SNP) is indicated above each set of bars. The error bars represent the standard error of the mean.

Nonsense-mediated decay (NMD) is a cellular process that degrades aberrant mRNAs, such as those with in-frame stop codons that encode truncated proteins. In *ERAP2*, NMD has been shown to degrade a rare mRNA form detected in a mantle-cell lymphoma that included an extra exon (after canonical exon 12) with an in-frame STOP codon [49]. The two stop codons present in exon 10 on Haplotype B also fulfill the established requirements for NMD [50]. Thus, the above-described absence of detectable truncated ERAP2 protein may be due to NMD of Haplotype B-derived mRNA. To test this hypothesis, we performed allele-specific quantitative real-time PCR (qRT-PCR) analysis of heterozygote LCLs under normal and NMD-inhibited conditions (by treating the cells with emetine, which blocks translation and NMD). We specifically examined the expression of three coding diagnostic SNPs (Figure 5A). All three SNPs showed significantly lower levels of *ERAP2* mRNA expressed from Haplotype B versus Haplotype A (Figure 5B) for all AB-heterozygote cell lines. Inhibition of NMD resulted in similar levels of *ERAP2* mRNA expression from Haplotypes A and B (Figure 5B). These data indicate that NMD acts on Haplotype B-derived *ERAP2* mRNA, accounting for both the reduced levels of Haplotype B-derived *ERAP2* cDNA and the absence of truncated ERAP2 protein.

### Effects of *ERAP2* variants on MHC class I presentation

**Figure 6:**
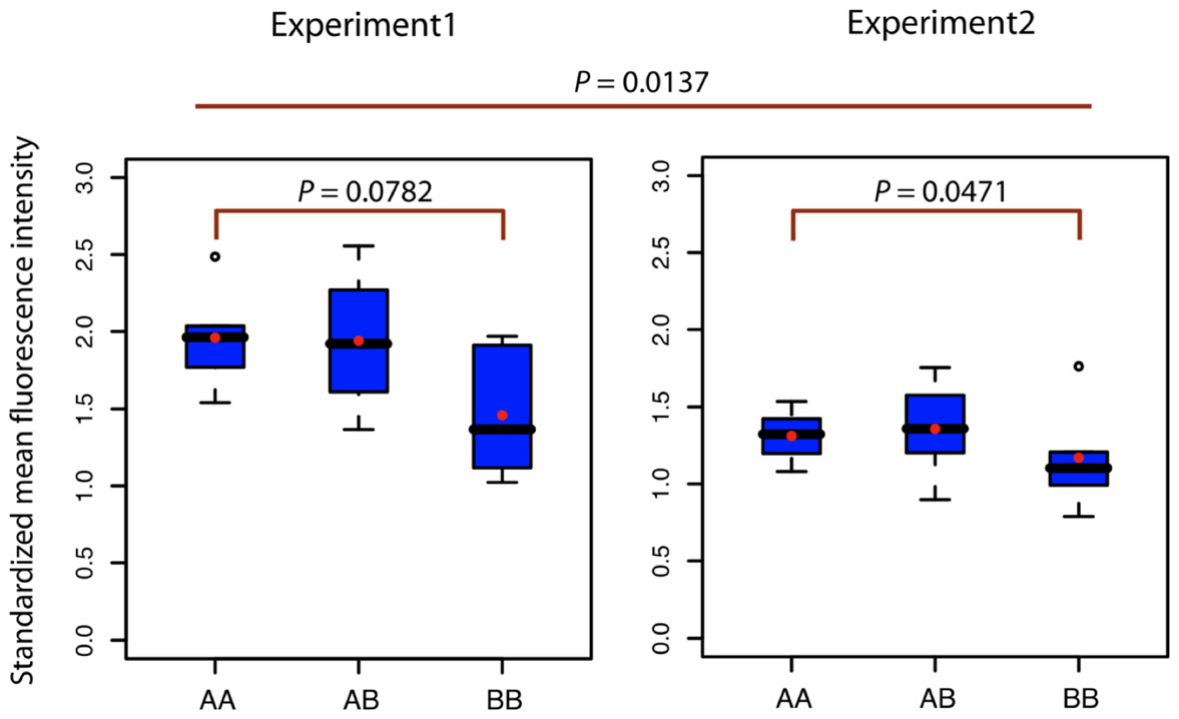
Standardized HLA-ABC mean fluorescence intensity of B-cells with various *ERAP2* genotypes. The distribution of observed levels of surface-expressed HLA-ABC for B cells of AA, AB, and BB individuals are graphically represented as boxplots (the blue box containing the 25th–75th percentile of the distribution, the black horizontal line indicating the median, the red dot reflecting the mean, and black circles representing outliers). Data are shown for two independent experiments (left and right). For each experiment, the significance level of the comparison between AA and BB homozygotes (T-test) is shown within the plot; the significance level of the effect of genotype in the global comparison between AA and BB homozygotes (two-way ANOVA) is shown above. A representative HLA-ABC fluorescence intensity plot is shown in Figure S7, and the mean fluorescence intensity boxplots of HLA-ABC and CD19 are presented in Figure S8.

Transient knock-down of *ERAP1* and *ERAP2* reduces the levels of MHC class I molecules on the surface of cultured cells [48]. To establish whether endogenous ERAP2 deficiency has a similar effect in BB-homozygotes, we examined the levels of MHC class I molecules on the surface of peripheral blood B cells by flow cytometry. Two experiments were performed to account for experimental variability. MHC class I (HLA-ABC) mean fluorescence intensities (MFIs) were lower on BB-homozygote cells compared to AA-homozygote cells; such a difference was not seen with CD19, a marker constitutively expressed by B cells (Figures S7 and S8). AB-heterozygotes showed a high level of variability (Figures S7 and S8). To account for the intrinsic variability among human samples, the HLA-ABC MFIs were standardized relative to CD19 (see Materials and Methods for details). Standardized HLA-ABC MFIs were also reduced in BB-homozygotes: a two-factor ANOVA showed that after controlling for differences among experiments (a significant factor, *P* = 0.0011), genotype significantly affects the level of standardized HLA-ABC MFIs (*P* = 0.0137). Such an effect is evident in both experiments (Figure 6), although the significance of the tests is reduced due to the smaller sample size (T-test: experiment 1, *P*-value = 0.0782; experiment 2, *P*-value = 0.0471). These results demonstrate that BB-homozygotes have reduced levels of MHC class I expression on B-cell surfaces.

### *ERAP1* evolution

In order to determine whether the signatures of selection seen with *ERAP2* are shared with its closely linked paralog (*ERAP1*), we analyzed the polymorphism data for *ERAP1* generated with our sample of 180 individuals. The SFS for *ERAP1* shows a slight enrichment in intermediate-frequency alleles (Figure 1C), which results in a significant departure from neutral expectations in the Yoruba, Palestinian, Han, and Toscani populations as measured by the MWUhigh test (Table 1). The Yoruba, Han, and Toscani populations also show departures from neutral expectations according to Tajima's D analysis (Table 1). *ERAP1* has 6.4 SNPs per fixed difference (45 coding SNPs and 7 coding fixed differences), a significant departure from neutral expectations (HKA test, Table 1). The estimated time to the most recent common ancestor of *ERAP1* variants is 2.84 Mya (standard deviation: 839,000 years).

The *ERAP1* haplotype network (Figure 2B) contains a large number of haplotypes, with a complex relationship among them and many reticulations that represent either recombination or recurrent mutation. In short, it does not reflect a highly structured haplotype network, likely due to the long-term effects of recombination. It is worth noting that LD between *ERAP1* and *ERAP2* is low (Figure S9), and the two most common *ERAP1* haplotypes do not show linkage with the two major *ERAP2* haplotypes (data not shown), indicating that the *ERAP1* signatures are independent from those of *ERAP2*. Additionally, we found no association between the *ERAP2* haplotypes and *ERAP1* splicing or expression differences (Text S1).

## Discussion

By generating and analyzing high-quality genome-sequence data, we have demonstrated that *ERAP2* has the distinct signatures of balancing selection that maintains intermediate-frequency alleles. These results validate our initial genome-wide findings [3], and indicate that the selective agent is not population-specific, because the detected signatures are similar among geographically diverse human groups. Selection has maintained *ERAP2* variants for an estimated 1.4 million years and, accordingly, the putatively selected variant rs2248374 is not polymorphic in chimpanzee (sequence analysis, n = 19) or orangutan (sequence analysis, n = 4), and no annotated chimpanzee SNP is shared with humans (dbSNP version 130). Interestingly, the derived allele was observed in a 4,000-year-old Paleo-Eskimo [51], showing that the non-functional ERAP2 form was present in ancient *Homo sapiens* populations. We are confident that the detected *ERAP2* genetic signatures are due to selection on the gene rather than on adjacent loci (e.g., *ERAP1* and *LNPEP*) because (a) signatures of balancing selection are tight in humans [3] due to the long-term effects of recombination [52], [53]; and (b) no linkage block shared between African, East Asian, and European HapMap populations links *ERAP2* with *ERAP1* or *LNPEP* (Figure S9).

*ERAP1* also shows signatures of selection, although the patterns are less dramatic than with *ERAP2*. The excess of polymorphism (over 7-fold compared with control regions) and subsequent high estimated coalescence time (2.8 Mya), combined with a modest enrichment in intermediate-frequency variants, suggest long-term balancing selection acting on *ERAP1*. Still, the gene lacks a striking excess of intermediate-frequency alleles as seen with *ERAP2*, and its haplotype network is not highly structured due to the long-term effects of recombination. Taken together, these results suggest that *ERAP1* has evolved under long-term balancing selection that either (1) maintains a large number of low-to-intermediate frequency variants; or (2) has changed, stopped, or weakened in recent evolutionary history.

*ERAP2* is particularly interesting due to the combination of its remarkable signatures of balancing selection and the pronounced functional differences between its two major haplotypes. Specifically, we showed that Haplotype A-derived mRNA encodes full-length, canonical ERAP2, while Haplotype B-derived mRNA undergoes differential splicing and NMD, resulting in undetectable levels of ERAP2. We studied LCLs, a particularly relevant cell type for MHC class I presentation. It is possible, though unlikely, that other tissues and/or developmental stages utilize alternate mechanisms that lead to the generation of ERAP2 protein from both haplotypes. Nevertheless, our data suggest that 25% of the population are AA homozygotes and generate abundant amounts of ERAP2 protein in lymphocytes, 50% are heterozygotes and generate reduced amounts of ERAP2 protein, and 25% are BB homozygotes and generate no or virtually no ERAP2 protein. Note that these frequencies are fairly consistent among all of the populations that we analyzed, as well as other human groups (Text S1). Therefore, based on our results, the *ERAP2* genotype should be accounted for in interpreting *ERAP2* studies, especially those focused on *ERAP2* expression and ERAP2 protein function. For instance, it may be interesting to reassess previous studies of ERAP2 that used immortalized or cancer cell lines and reported contradictory results (Text S1).

In light of the differences in *ERAP2* expression from the A versus B haplotypes, what are the biological consequences of lower ERAP2 protein levels in AB and BB individuals? The evidence that ERAP2 has a functional role in humans is both experimental [24], [48] and evolutionary (i.e., the level of constraint of *ERAP2* in humans is similar to that in other mammals; Tables S2 and S3 and Text S1). *ERAP1* and *ERAP2* share 51% sequence identity [18], and their protein products can form heterodimers [48], though the functional nature of these dimmers remains elusive. While both ERAP1 and ERAP2 act as aminopeptidases, there are important differences in their peptide specificity [48]; for example, specific residues in the HIV-derived peptides R10L (from the HIV-*gag* protein) and K51I (from the HIV-*env* protein) are preferentially trimmed by ERAP2 [15], [48]. ERAP1 and ERAP2 likely act in a concerted fashion to provide important protein-trimming activity in the human endoplasmic reticulum, with each differentially contributing to the pool of antigenic peptides [15].

A possible effect of ERAP2 deficiency could be an alteration in the set of peptides available for the MHC. For example, mouse studies have shown that knocking out *ERAP1* results in alterations in the set of presented epitopes [54]–[56] and immunodominance hierarchy [57]. These changes ultimately influence T-cell response [58]. Remarkably, HIV evolves to avoid ERAP1 trimming [59], suggesting that despite high redundancy in MHC class I presentation of proteins, the particular presented epitope (which is highly dependent on antigen processing [60]) influences immune response. The absence of *ERAP2* in the mouse genome precludes performing similar knock-out studies as with *ERAP1*, although one could envision a similar effect of ERAP2 deficiency in antigen presentation. Importantly, this alteration in the set of presented epitopes may have a previously unrecognized influence, for example, on immunological function, auto-immunity, and histocompatibility.

In addition to these putative differences, we demonstrated that ERAP2 deficiency results in a quantitative reduction of MHC class I levels. Specifically, we found significantly less MHC class I on the surface of B cells from BB-homozygotes compared to AA-homozygotes. This result is consistent with the reduced MHC class I cell-surface expression observed after transient knock-down of *ERAP1* or *ERAP2* in cultured cells [48], the reduced MHC class I cell-surface expression seen in *ERAP1*-knock-out mice [54]–[56], [61], and our observation that *ERAP1* is not upregulated to compensate for ERAP2 deficiency in cells from BB-homozygotes (Text S1). The reduced MHC class I cell-surface expression might be due to reduced stability of the MHC complex when loaded with suboptimal peptides, as has been suggested with ERAP1-deficient mice [55], [56], [62].

Because we studied a natural deficiency of ERAP2, our results suggest that the observed reduction in MHC class I levels is not transient and that BB-homozygotes likely have lower background levels of MHC presentation. The effect of *ERAP2* knock-down is not evident when the antigen-processing machinery is activated by IFN-γ [48], consistent with the results with *ERAP1* knock-out mice [55] (but see [63]). This suggests that rather than affecting inflammatory response, ERAP2 deficiency might be relevant to basal MHC class I presentation. Antigen processing is an inefficient process, with an estimated 10,000 proteins degraded to form a single MHC-peptide complex [64]. Therefore, reduced MHC class I levels may result in a lower presentation of rare antigens (particularly, in this case, of those preferentially trimmed by ERAP2), possibly delaying their specific immune response. Further studies that correlate *ERAP2* genotype with levels of MHC class I expression in other tissues, and with the presentation and recognition of specific antigens, are needed to more clearly define the influence of ERAP2 deficiency on immune response.

An important remaining question is what selective mechanism accounts for the maintenance of a decayed form of *ERAP2*. Selection of polymorphic truncating variants is not unusual, with notable examples in domesticated species [65], [66] and natural populations [67]–[69]. ERAP2 is involved in a variety of biological processes, including immunity, inflammation, and, perhaps, the regulation of blood pressure; it has also been linked to pathologies such as pre-eclampsia (see Introduction). Therefore, a number of mechanisms may explain the balancing selection seen with *ERAP2*. Overdominance is probably the most widely considered mechanism for balancing selection. In this case, overdominance could be explained if heterozygotes had the optimal level of ERAP2 protein. This would be unlikely if MHC levels are the selected phenotype, because MHC cell-surface expression is variable in heterozygotes (Figure 6). Regardless, AB-heterozygotes might have a different epitope hierarchy than AA or BB homozygotes that account for the putative selective advantage.

Another possible mechanism is oscillating selection, where alternative genotypes are advantageous at different times. This has been proposed for *FLT1*, a gene that, like *ERAP2*, is associated with pre-eclampsia [70]. The short alleles of the *FLT1* repetitive region are deleterious during malaria season but appear to be beneficial out of malaria season. There is no known link between malaria and *ERAP2* genotypes, and the signatures of selection are observed in non-malaria-suffering regions. However, one can imagine other scenarios where seasonal agents could favor the AA or BB genotype at different times, with adequate temporal fluctuation and selective coefficients to maintain both alleles in the population.

Another interesting mechanism of balancing selection is pleiotropic selection, where different genotypes are advantageous for different biological processes. This has been suggested as an explanation for the highly polymorphic *KIR* loci [12], with *KIR* A haplotypes protecting against hepatitis C virus infection but being a risk factor for pre-eclampsia. In this model, differential selection between an immunological function and reproduction maintains genetic diversity. Interestingly, a recent study revealed an association between the *ERAP2* Haplotype A and pre-eclampsia in an Australian cohort [34]. The presence of functional ERAP2 and the resulting high levels of MHC class I may be beneficial in some situations (e.g., in response to tumors or pathogens) yet detrimental in others (e.g., in the case of auto-immunity).

Immune-related genes are subject to natural selection in humans [71]–[74], although the relative importance of positive and balancing selection is not fully defined (reviewed in [75]). In the case of MHC class I presentation, the elements responsible for recognition and presentation of antigenic peptides have evolved under balancing selection [4]–[6], [10]–[12], as have the two genes that encode the enzymes responsible for the final trimming of antigenic peptides. The *ERAP2* genetic diversity identified here has biological implications in terms of influencing the levels of MHC class I on the cell surface and likely downstream antigen presentation. Future studies should help to establish the influence that this genetic variation has on other biological processes, such as immunocompetence, histocompatibility, regulation of blood pressure, and risk to immune-related disorders such as auto-immunity and pre-eclampsia.

## Materials and Methods

### Ethics statement

Anonymized samples for this study were derived from allogeneic blood donor samples that already existed and would otherwise be discarded. As the samples were provided anonymously, the NIH Office Of Human Subjects Research approved the use of these samples on an exemption basis, per federal code (45CFR46), without the need for IRB review or informed consent.

### Sequence generation

The complete *ERAP2* coding region and some exon-adjacent intronic regions (8794 bp total, 2883 bp of which are protein coding) were sequenced in 180 individuals from 6 geographically diverse human groups. Specifically, we studied 30 individuals from each of the following HapMap [76] populations: Yoruba (Nigeria), Luhya (Kenya), Gujarati Indians (living in Houston, TX, USA), Han (China), and Toscani (Italy). As a representative Middle Eastern population, we also studied 30 Palestinian (Israel) individuals from the National Laboratory for the Genetics of Israeli Populations (Tel-Aviv University). The same 180 individuals were also used for sequencing portions of the *ERAP1* gene (9753 bp total, 2847 bp of coding sequence). The regions sequenced are shown in Figure S1.

Regions of interest were PCR-amplified and sequenced (bidirectional Sanger-based sequencing), and SNPs were detected with Polyphred/Polyphrap. To minimize sequencing errors, variants residing within the first and last 50 bp of each amplified segment were discarded. Additionally, we manually reviewed all variants associated with discordant results between overlapping amplimers, variants with a quality score lower than 99, singletons, and triallelic SNPs. The ancestry of each SNP was inferred through comparison with the chimpanzee, orangutan, and macaque genome sequences [77], [78, genome.ucsc.edu]. Fixed differences with chimpanzee were identified by comparison with the chimpanzee genome sequence [77].

As a proxy for neutrality, we sequenced 47 control regions. Such regions consisted of unlinked, ancient processed pseudogenes that do not encode a functional protein and are thus expected to evolve in a neutral fashion. The control regions are not part of gene families, are far from genes, do not overlap putative functional elements, are conserved as pseudogenes in chimpanzees, orangutans, and macaques, and have recombination rates and GC contents similar to coding genes. Details about these control regions can be found in the Text S1.

### Evolutionary analysis

The generated sequence data were analyzed using three neutrality tests: MWUhigh, Tajima's D, and HKA. MWUhigh [37] compares the SFS of a region of interest with the SFS of a neutral region(s) (e.g., control regions) to determine whether the former is consistent with neutral expectations [37]. Specifically, we applied MWUhigh to the folded SFS, which becomes significant only in the case of an excess of intermediate-frequency alleles [3]. Tajima's D [38] compares two estimates of θ (the scaled mutation rate) and, when significantly positive, identifies genealogies with long internal branches consistent with long-term balancing selection. Finally, HKA [39] identifies regions with an unusual density of polymorphisms when compared with divergence and with the patterns of neutral loci. For the HKA test, we focused only on coding regions and used the chimpanzee as an outgroup. MWUhigh was calculated using an in-house C script, while Tajima's D and HKA were calculated using libsequence [79].

The significance of all neutrality tests was assessed by 10,000 coalescent simulations with ms [80]. Selecting an appropriate demographic model for the simulations is crucial to avoid spurious detection of signatures of selection. Our null model followed a recently published demographic scenario that included African, Asian, and European populations [81] and that was a better fit to our control data than previously proposed demographic models. The divergence to chimpanzee was adjusted in the simulations to fit the ratio of SNPs to fixed differences of the control regions. Simulations were conditioned on the total number of informative sites, and the recombination rate was set to 10^−6^ per base pair, the estimated recombination rate of this genomic region (genome.ucsc.edu). All analyses were performed with an in-house PERL program (Neutrality Test Pipeline).

Haplotypes of the coding SNPs were inferred using PHASE [82], and the haplotype network was created with Network [83]. The estimated age of the haplotypes was calculated using Network and calibrated with chimpanzee, considering a divergence time of 6 Mya.

### Analysis of splicing

We analyzed the *ERAP2* cDNA from LCLs of HapMap Yoruba individuals with different genotypes: AA-homozygotes (GM18504, GM18505, GM11832, and GM07000), BB-homozygotes (GM18507, GM19240, GM12891, and GM12892), or AB-heterozygotes (GM18861, GM18870, GM19137, and GM19201). The cell lines were obtained from the Corriell Cell Repositories (ccr.coriell.org). Total RNA was isolated from each cell line using Trizol reagent (Invitrogen) and the RNeasy miniprep kit (Qiagen). cDNA was synthesized from 1 µg of total RNA using the Superscript III First Strand Reverse Transcriptase Kit and random hexamers (Invitrogen). The *ERAP2* full-length transcript (exons 1 to 19) was amplified using Expand High Fidelity PCR System (Roche) from cDNA prepared from LCLs that were AA-homozygote (GM18504) or BB-homozygote (GM18508). These PCR products were cloned into the pCR4-TOPO vector (Invitrogen) and at least six clones for each haplotype were sequenced (3100 Genetic Analyzer, Applied Biosystems). Primer sequences for this experiment and for the exon 10 splice-variant screening can be found in Table S4.

The effect of rs2248374 on *ERAP2* mRNA splicing was assessed using two *in silico* methods. First, we used GeneID [46] to predict the splicing of mRNA derived from the two haplotypes (Text S1). Second, we used MaxEnt [47] to predict the splicing potential of the constitutive splice site with: (1) the A allele: ATGGTAAGG; and (2) the G allele: ATGGTGAGG.

### Western blot analyses

Western blot analysis was performed as previously described [84]. Briefly, protein extracts from approximately 3×10^3^ cells were separated on a 4–12% NuPage Bis-Tris gel (Invitrogen) at 125 V for 100 minutes in 1× NuPage MES SDS Running Buffer (Invitrogen). After transfer to a nitrocellulose membrane, proteins were detected using a 1∶5,000 dilution of primary antibody [goat anti-ERAP2 polyclonal antibody (AF3830, R&D Systems) and mouse anti-ERAP2 polyclonal (ab69037, Abcam); anti-ß-actin monoclonal prepared in mouse (A5316, Sigma)] and a 1∶10,000 dilution of secondary antibody conjugated with horseradish peroxidase (HRP) [goat anti-mouse IgG (sc-2005; Santa Cruz Biotechnology) and donkey anti-goat IgG (sc-2020; Santa Cruz Biotechnology)]. Proteins were then visualized by autoradiography after treatment with substrate to HRP (Thermo Scientific) for 5 minutes. The ratio of the intensity of the full-length ERAP2 band of AA-homozygotes to AB-heterozygotes was calculated using ImageJ (rsbweb.nih.gov/ij/index.html).

### Analysis of allele-specific gene expression

AB-heterozygote LCLs were treated with 100 µg/ml of emetine (Sigma) for 7 hours to inhibit NMD [50]. Parallel cultures were left untreated and grown at standard conditions. Total RNA was prepared from each cell line and used to generate cDNA as described earlier. We quantified haplotype-specific *ERAP2* cDNA in triplicate using an allele-discriminating TaqMan genotyping assay for three coding diagnostic SNPs (C_3282749_20 for rs2549782, C_25649530_10 for rs2548538, and C_25649516_10 for rs2287988; Applied Biosystems) as previously described [85]. Briefly, for each allele-specific assay, we generated a standard curve consisting of serial dilutions of two HapMap genomic DNA samples homozygous for either the Haplotype A (GM18504) or Haplotype B (GM18508) allele. We used a heterozygous genomic DNA sample (GM18861) to validate the regression equation, in which we expect to see a mean allelic ratio of 1.0 since both the Haplotype-A and Haplotype-B alleles are present in an equal proportion.

### HLA expression on B-cell surface

Two experiments (labeled 1 and 2 in Figure 6) were performed with 16 samples each. Human peripheral blood mononuclear cells (PBMCs) were isolated from buffy coats using a Ficoll/Histopaque gradient (Lonza). PBMCs were washed and cultured using RPMI 1640 supplemented with 10% fetal calf serum, 1% penicillin and streptomycin, 0.2 M L-glutamine, and 20 mM Hepes. Surface staining was measured by flow cytometry using fluorescence-labeled antibodies specific to CD19 (labeled with APC; clone HIB19; eBioscience) and HLA-ABC (labeled with FITC; clone W6/32; eBioscience) which reacts to HLA-A, B, and C. Flow-cytometry data analysis was performed with Flojo software (Treestar). Specifically, we measured HLA-ABC MFIs from a population of B cells gated by CD19 (a constitutive B-cell marker) intensity. Gating and analysis were carried out blindly with respect to genotypes. In order to standardize HLA-ABC MFI in light of the intrinsic variability among human samples, a standardized HLA-ABC measure was calculated for each sample by dividing the HLA-ABC MFI by the CD19 MFI for each sample. The values were partitioned by experiment and sub-partitioned by genotype; within each of these groups, outliers were removed (defined as samples with values under or over 1.5-times the inter-quartile range). It is worth noting that the inclusion of outliers did not affect the results. Two sets of analyses were performed for each of these three measures (HLA-ABC, CD19, and standardized HLA-ABC) as the dependent variable. First, a T-test was used to detect differences between cells with AA and BB genotypes for each experiment. Second, a two-factor ANOVA was performed for each measure using the data generated with all AA or BB samples, where the two factors of the ANOVA were genotype and experiment. Genotyping was performed by PCR amplification and sequencing of DNA prepared from the PBMCs (DNeasy Blood and Tissue kit, Qiagen) using primers flanking rs2248374 (see Table S4 for primer sequences).

## Supporting Information

Figure S1 Genomic regions sequenced. Chromosomal position and gene structure of *ERAP1* and *ERAP2* genes. The green boxes above the gene structures mark the regions sequenced.
(0.25 MB TIF)

Figure S2 Allele site-frequency spectrum (SFS) of *ERAP2*, control regions, and *ERAP1* in each population when only coding SNPs are considered for *ERAP2* and *ERAP1*. The X-axis reflects the absolute frequency of the derived allele, while the Y-axis reflects the frequency of that allele frequency bin in the generated dataset. To account for missing data, the frequencies were projected to a sample size of 15 chromosomes [Nielsen R, Hubisz MJ, Clark AG (2004) Reconstituting the frequency spectrum of ascertained single-nucleotide polymorphism data. Genetics 168: 2373–2382]. See the SFS of all SNPs in Figure 1.
(1.12 MB TIF)

Figure S3 Integrated haplotype score (iHS) test display in each HapMap population. The graphs show an ordered display of the haplotypes in the core genomic region (*ERAP2*), located in the center. The ancestral allele is represented in blue, and the derived allele in red. Color switches mark a transition to a different haplotype (haplotter.uchicago.edu).
(1.30 MB TIF)

Figure S4 Immunoblot analyses of ERAP2 using mouse mAb 3F5 antibody of protein extracted from cell lines. 50 µg of protein extracted from various human cell types [LCLs of each *ERAP2* genotype (AA, AB, and BB), a neuronal cell line (SHSY5Y), an embryonic kidney cell line (HEK293T), and a cervical cancer cell line (HELA)] were tested for ERAP2 protein using primary mouse mAb 3F5 [Saveanu L, Carroll O, Lindo V, Del Val M, Lopez D, et al. (2005) Concerted peptide trimming by human ERAP1 and ERAP2 aminopeptidase complexes in the endoplasmic reticulum. Nat Immunol 6: 689–697] in the following concentration: A, 0.5 µg/ml; B, 0.125 µg/ml. Full-length ERAP2 is expected at approximately 120 kDa, while the putative truncated form of ERAP2 is expected at approximately 60 kDa. Note the reduced levels of full-length ERAP2 in SHSY5Y, HEK293T, and HELA.
(4.96 MB TIF)

Figure S5 Haplotype network of *ERAP2* with both coding and non-coding SNPs. Circles represent haplotypes, with the areas proportional to the frequency of the haplotype (color-coded by population). The lines connecting the haplotypes have a length proportional to the number of mutations that differentiate the two haplotypes. Reticulations reflect recombinations or recurrent mutations. The ancestral state was inferred using the chimpanzee sequence data.
(1.34 MB TIF)

Figure S6 Haplotype network of *ERAP2* with chimpanzee. Circles represent haplotypes, with the areas proportional to the frequency of the haplotype (color-coded by population). The lines connecting the haplotypes have a length proportional to the number of mutations that differentiate the two haplotypes. Reticulations reflect recombinations or recurrent mutations. The chimpanzee sequence represents the reference chimpanzee genome sequence for *ERAP2*.
(0.85 MB TIF)

Figure S7 HLA-ABC fluorescence intensity of representative samples with *ERAP2* AA and BB genotypes.
(0.34 MB TIF)

Figure S8 HLA-ABC and CD19 mean fluorescence intensities of B cells with various *ERAP2* genotypes. The distribution of observed levels of surface-expressed HLA-ABC for B cells with AA, AB, and BB genotypes are graphically represented as boxplots (the blue box containing the 25th–75th percentile of the distribution, the black horizontal line indicating the median, the red dot reflecting the mean, and black circles representing outliers). HLA-ABC results are shown on the left, and CD19 results are shown on the right. Data are shown for two independent experiments (left and right in each case). For each experiment, the significance level of the comparison between AA and BB homozygotes (T-test) is shown within the plot; the significance level of the effect of genotype in the global comparison between AA and BB homozygotes (two-way ANOVA) is shown below.
(0.27 MB TIF)

Figure S9 Linkage disequilibrium (LD) in the *ERAP1*, *ERAP2*, *LNPEP* genomic region based on HapMap polymorphism data. The strength of LD between a pair of SNPs is shown by the color of the diamond found at the intersection point connecting them: LD decreases from red to pink to blue to white (genome.ucsc.edu). YRI represents the Yoruba population, CEU the CEPH European sample, and ASN the Han Chinese and Japanese HapMap populations.
(22.86 MB TIF)

Table S1 Summary statistics and neutrality tests. S: number of SNPs; TajD: Tajima's D; p(TajD): *P*-value for Tajima's D test; p(MWU): *P*-value for MWUhigh test; FixedDiff: number of fixed differences with chimpanzee; p(HKA): *P*-value for HKA test.
(0.10 MB DOC)

Table S2 *dN*/*dS* of *ERAP2* and *ERAP1*. Estimated *dN*/*dS* ratios for the model that infers a single ratio for the whole phylogeny (*Complete phylogeny*) and estimated terminal branch *dN*/*dS* for the model that allows free ratios among branches (*Lineage-specific*). Dashes indicate species that lack the gene, while dots indicate species for which sequence could not be obtained. Likelihood ratio test results for the different analyses performed are in Table S3.
(0.03 MB DOCX)

Table S3 Models of evolution used for analyzing *ERAP2* and *ERAP1*. *P*-values of the log likelihood ratio test for all model comparisons performed (see Text S1).
(0.04 MB DOC)

Table S4 PCR primers.
(0.04 MB DOC)

Text S1 Supporting materials.
(0.11 MB DOC)
