# Supplementary figures and images for: Balancing Selection Maintains a Form of ERAP2 that Undergoes Nonsense-Mediated Decay and Affects Antigen Presentation
Source: PLoS Genet. 2010 Oct 14;6(10):e1001157. doi: 10.1371/journal.pgen.1001157 (PMC2954825; doi:10.1371/journal.pgen.1001157)

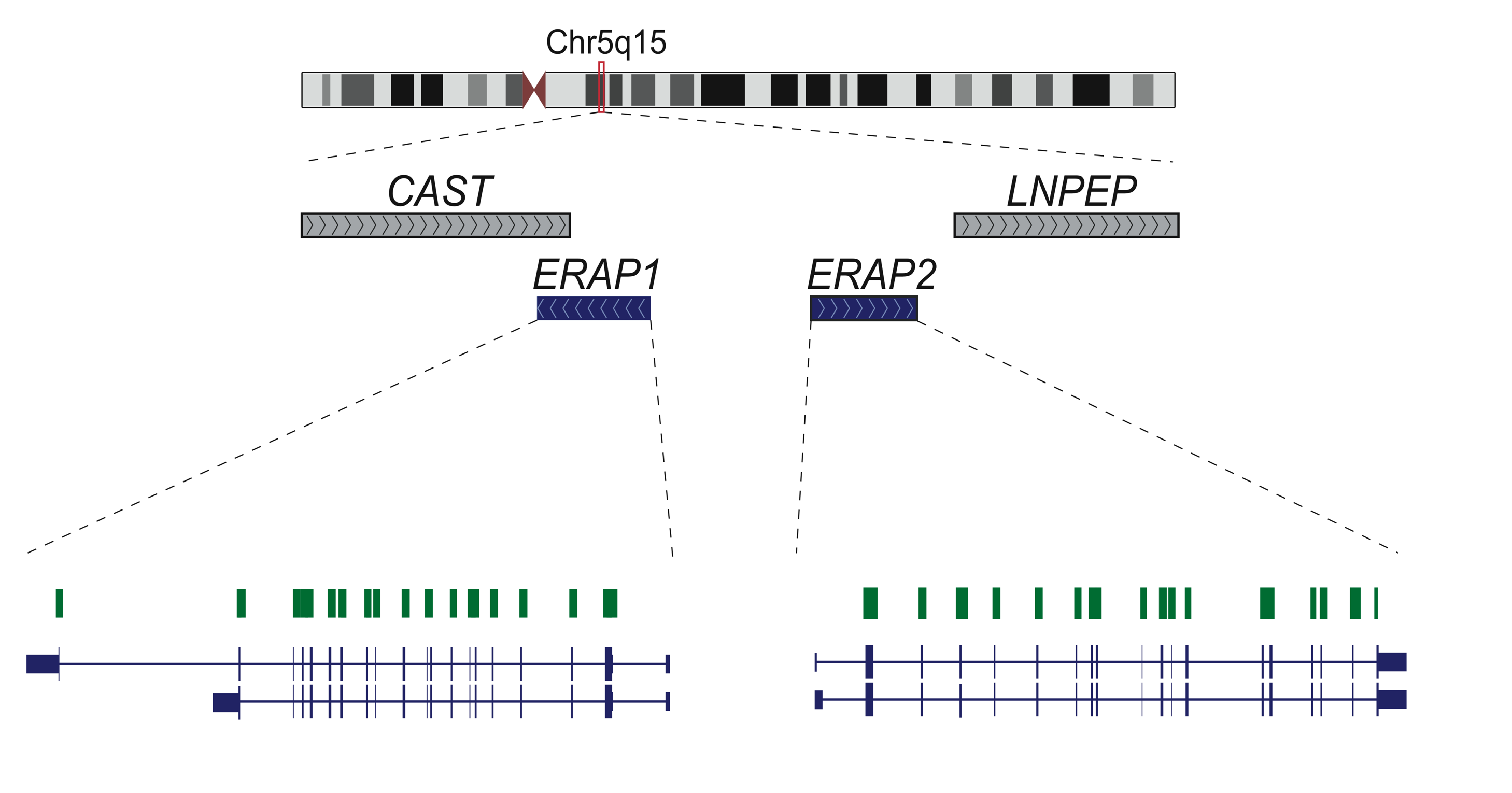

Supplement: Figure S1 — Genomic regions sequenced. Chromosomal position and gene structure of ERAP1 and ERAP2 genes. The green boxes above the gene structures mark the regions sequenced. (0.25 MB TIF) [file pgen.1001157.s001.tif]

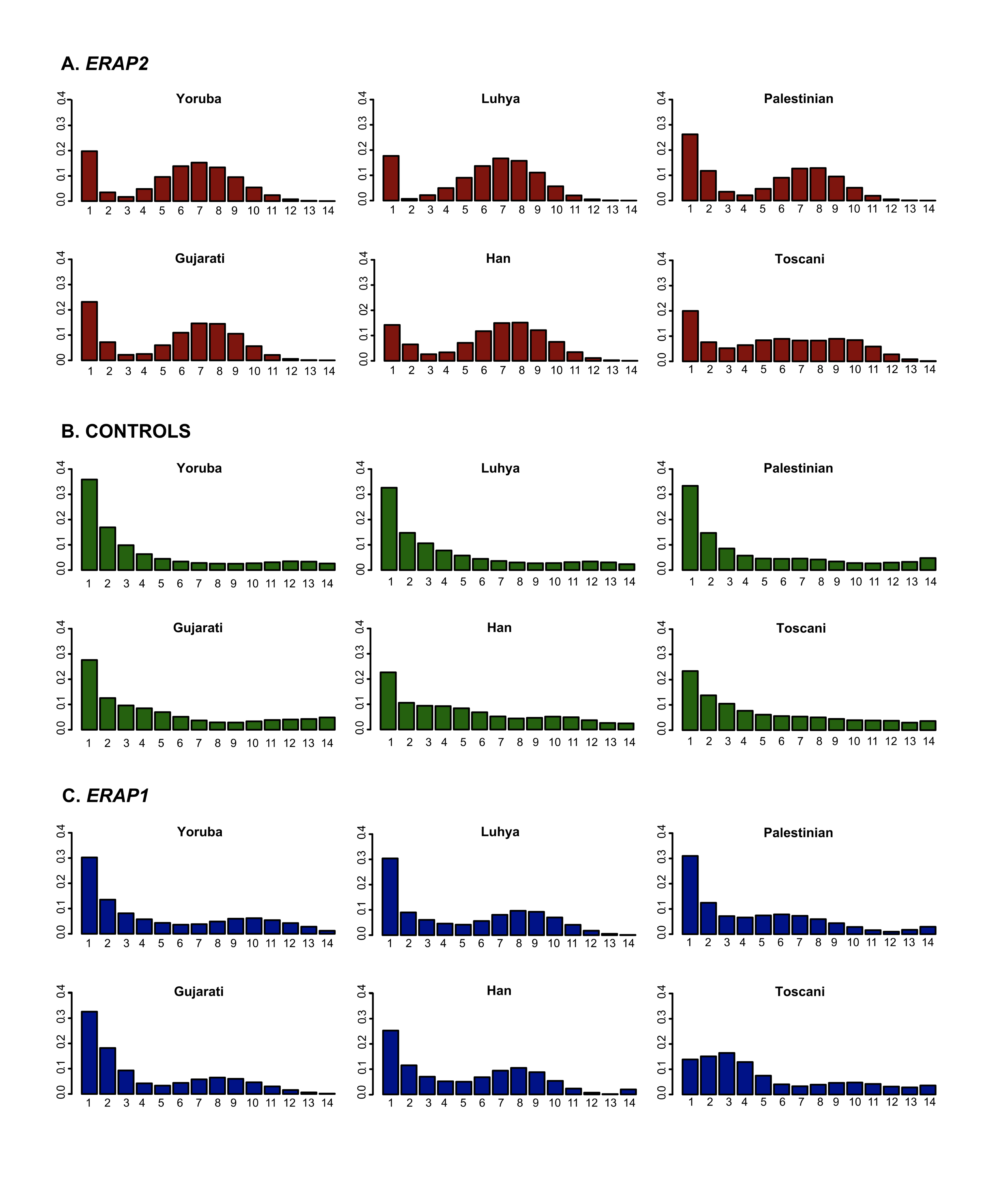

Supplement: Figure S2 — Allele site-frequency spectrum (SFS) of ERAP2, control regions, and ERAP1 in each population when only coding SNPs are considered for ERAP2 and ERAP1. The X-axis reflects the absolute frequency of the derived allele, while the Y-axis reflects the frequency of that allele frequency bin in the generated dataset. To account for missing data, the frequencies were projected to a sample size of 15 chromosomes [Nielsen R, Hubisz MJ, Clark AG (2004) Reconstituting the frequency spectrum of ascertained single-nucleotide polymorphism data. Genetics 168: 2373–2382]. See the SFS of all SNPs in Figure 1. (1.12 MB TIF) [file pgen.1001157.s002.tif]

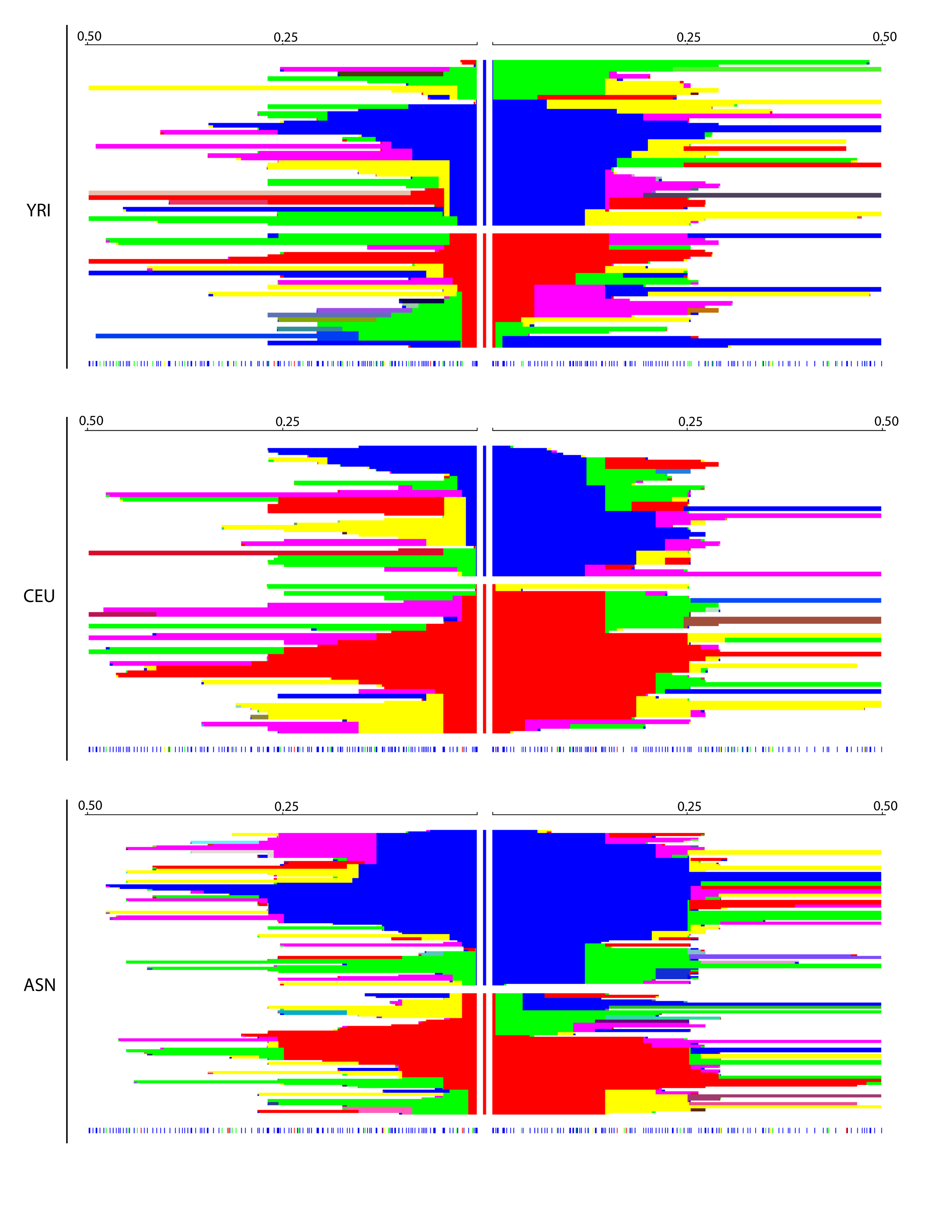

Supplement: Figure S3 — Integrated haplotype score (iHS) test display in each HapMap population. The graphs show an ordered display of the haplotypes in the core genomic region (ERAP2), located in the center. The ancestral allele is represented in blue, and the derived allele in red. Color switches mark a transition to a different haplotype (haplotter.uchicago.edu). (1.30 MB TIF) [file pgen.1001157.s003.tif]

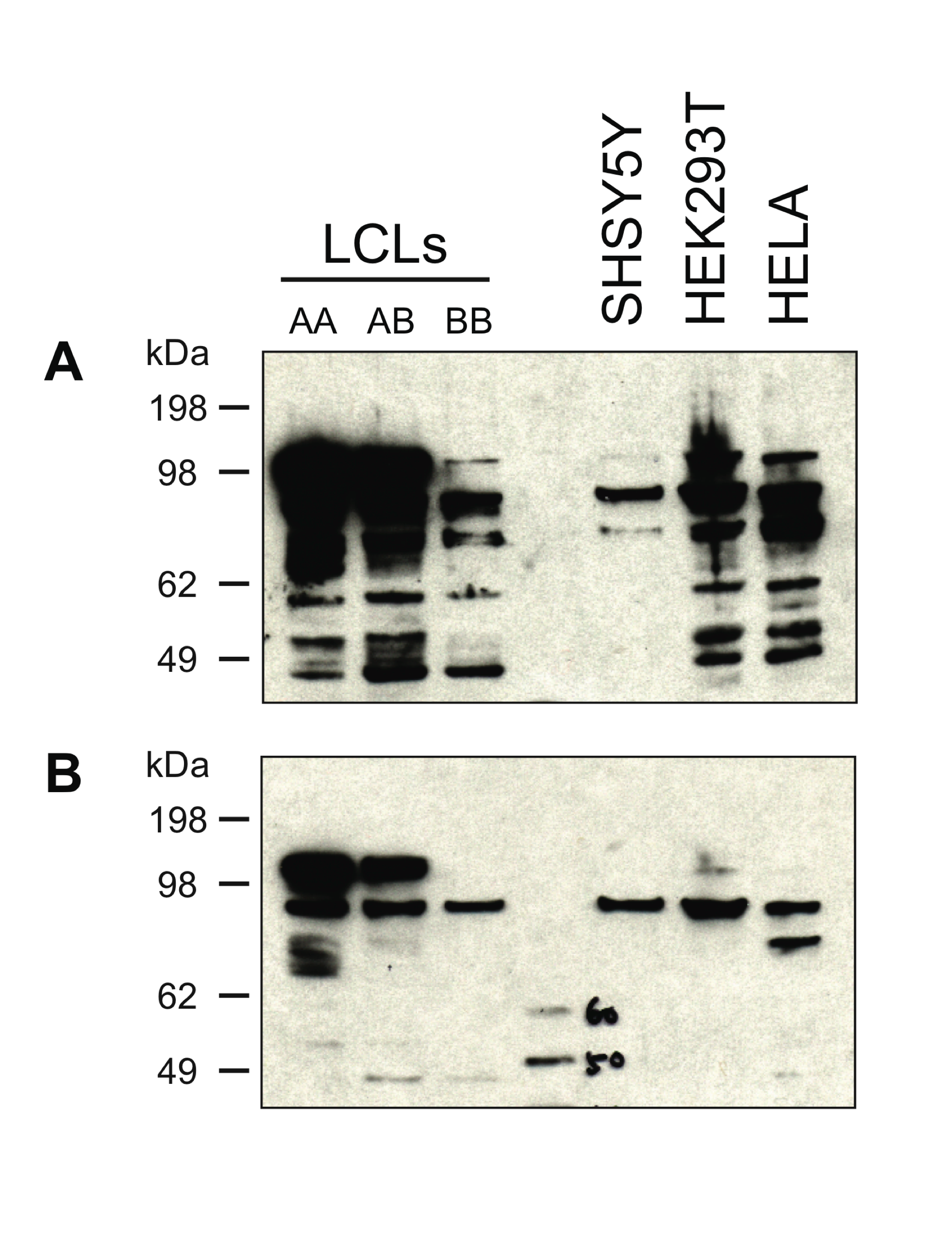

Supplement: Figure S4 — Immunoblot analyses of ERAP2 using mouse mAb 3F5 antibody of protein extracted from cell lines. 50 µg of protein extracted from various human cell types [LCLs of each ERAP2 genotype (AA, AB, and BB), a neuronal cell line (SHSY5Y), an embryonic kidney cell line (HEK293T), and a cervical cancer cell line (HELA)] were tested for ERAP2 protein using primary mouse mAb 3F5 [Saveanu L, Carroll O, Lindo V, Del Val M, Lopez D, et al. (2005) Concerted peptide trimming by human ERAP1 and ERAP2 aminopeptidase complexes in the endoplasmic reticulum. Nat Immunol 6: 689–697] in the following concentration: A, 0.5 µg/ml; B, 0.125 µg/ml. Full-length ERAP2 is expected at approximately 120 kDa, while the putative truncated form of ERAP2 is expected at approximately 60 kDa. Note the reduced levels of full-length ERAP2 in SHSY5Y, HEK293T, and HELA. (4.96 MB TIF) [file pgen.1001157.s004.tif]

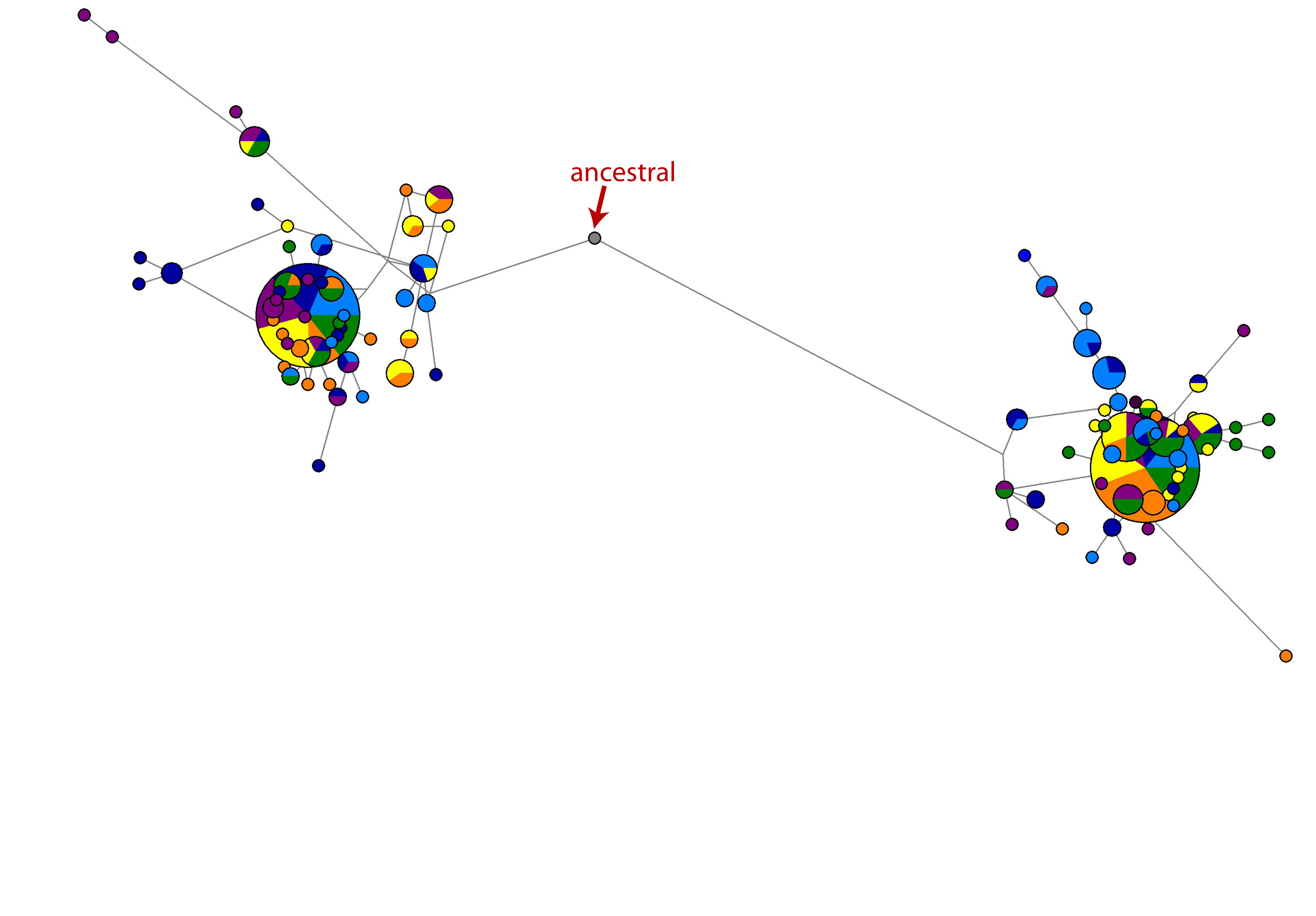

Supplement: Figure S5 — Haplotype network of ERAP2 with both coding and non-coding SNPs. Circles represent haplotypes, with the areas proportional to the frequency of the haplotype (color-coded by population). The lines connecting the haplotypes have a length proportional to the number of mutations that differentiate the two haplotypes. Reticulations reflect recombinations or recurrent mutations. The ancestral state was inferred using the chimpanzee sequence data. (1.34 MB TIF) [file pgen.1001157.s005.tif]

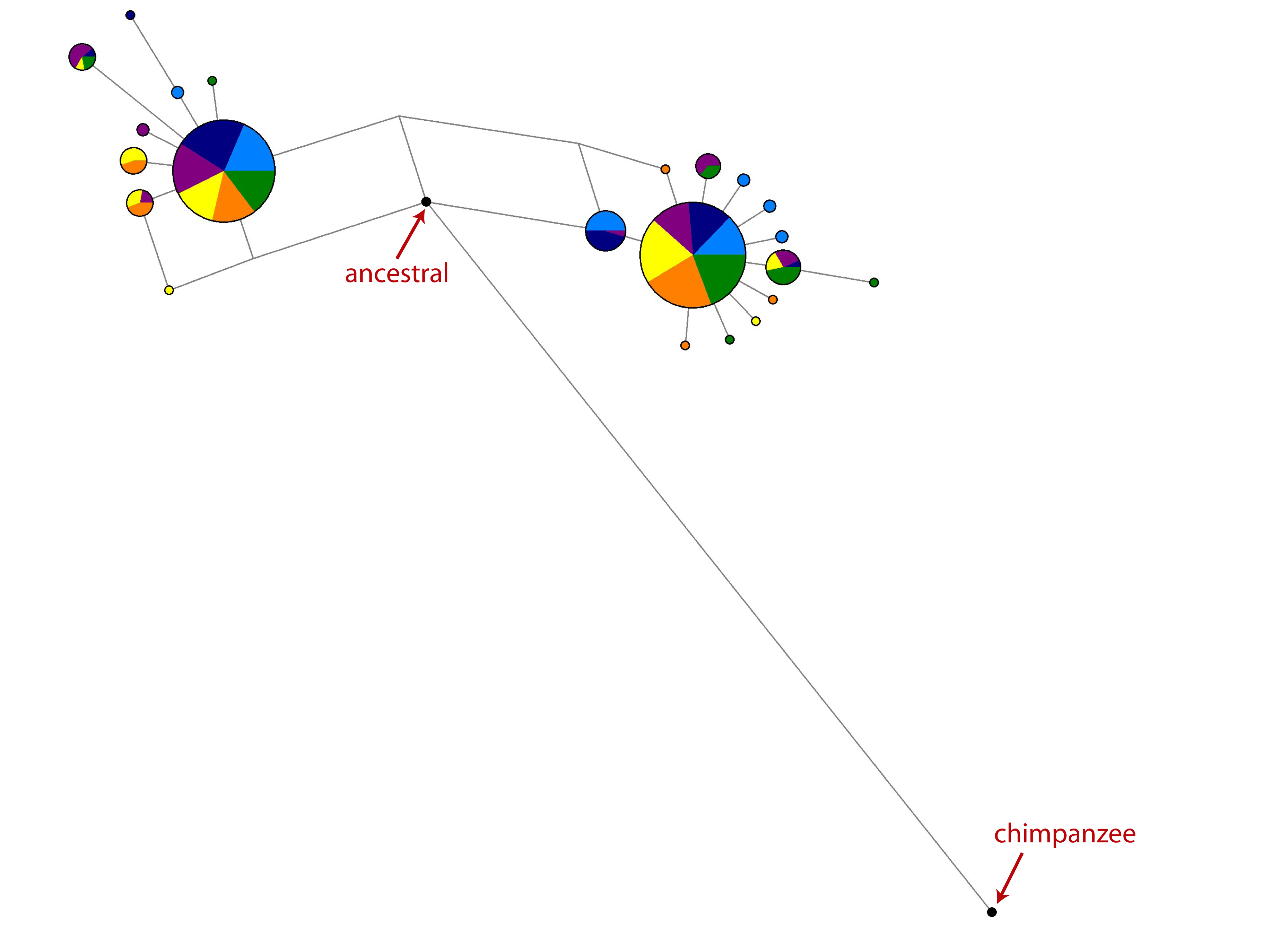

Supplement: Figure S6 — Haplotype network of ERAP2 with chimpanzee. Circles represent haplotypes, with the areas proportional to the frequency of the haplotype (color-coded by population). The lines connecting the haplotypes have a length proportional to the number of mutations that differentiate the two haplotypes. Reticulations reflect recombinations or recurrent mutations. The chimpanzee sequence represents the reference chimpanzee genome sequence for ERAP2. (0.85 MB TIF) [file pgen.1001157.s006.tif]

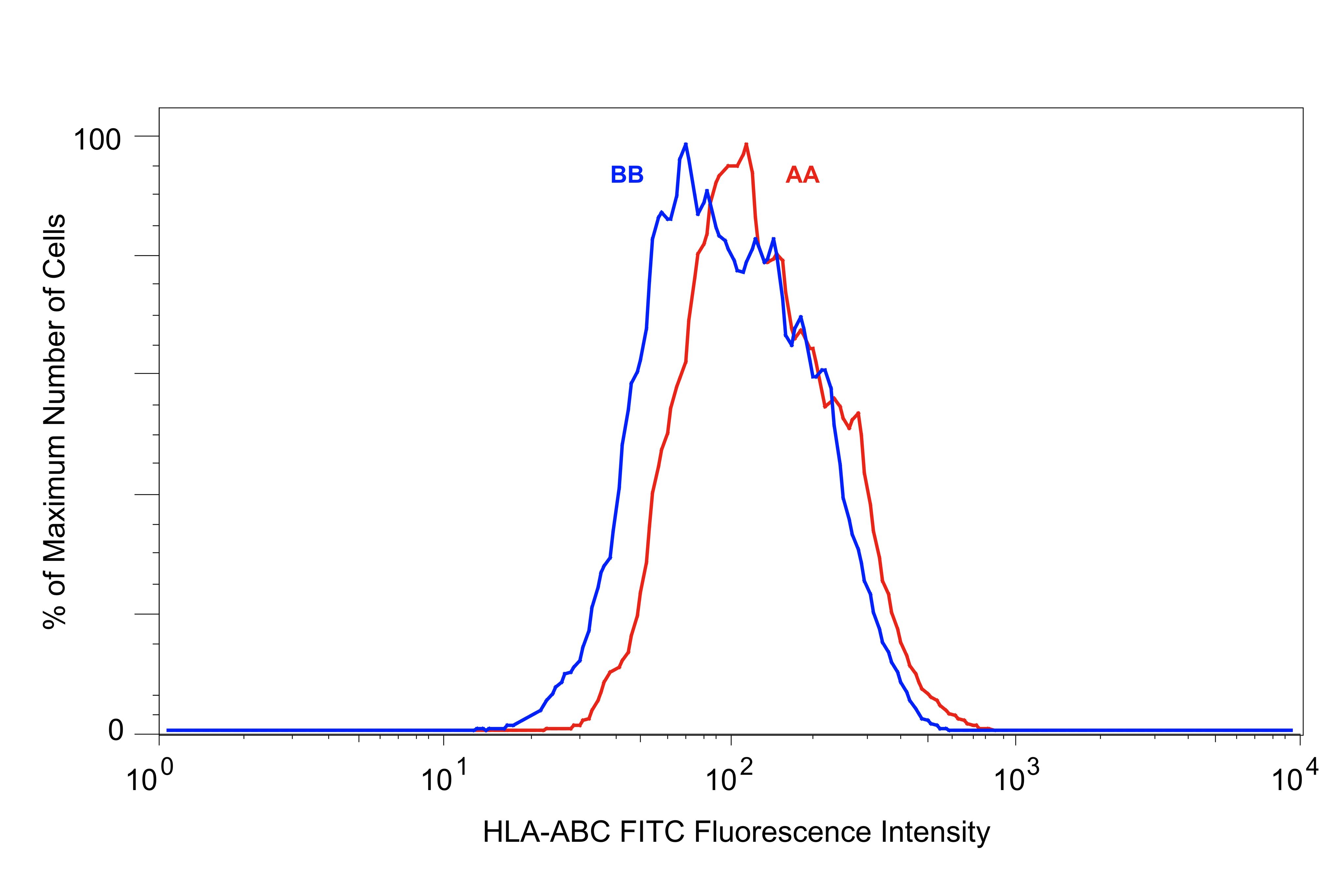

Supplement: Figure S7 — HLA-ABC fluorescence intensity of representative samples with ERAP2 AA and BB genotypes. (0.34 MB TIF) [file pgen.1001157.s007.tif]

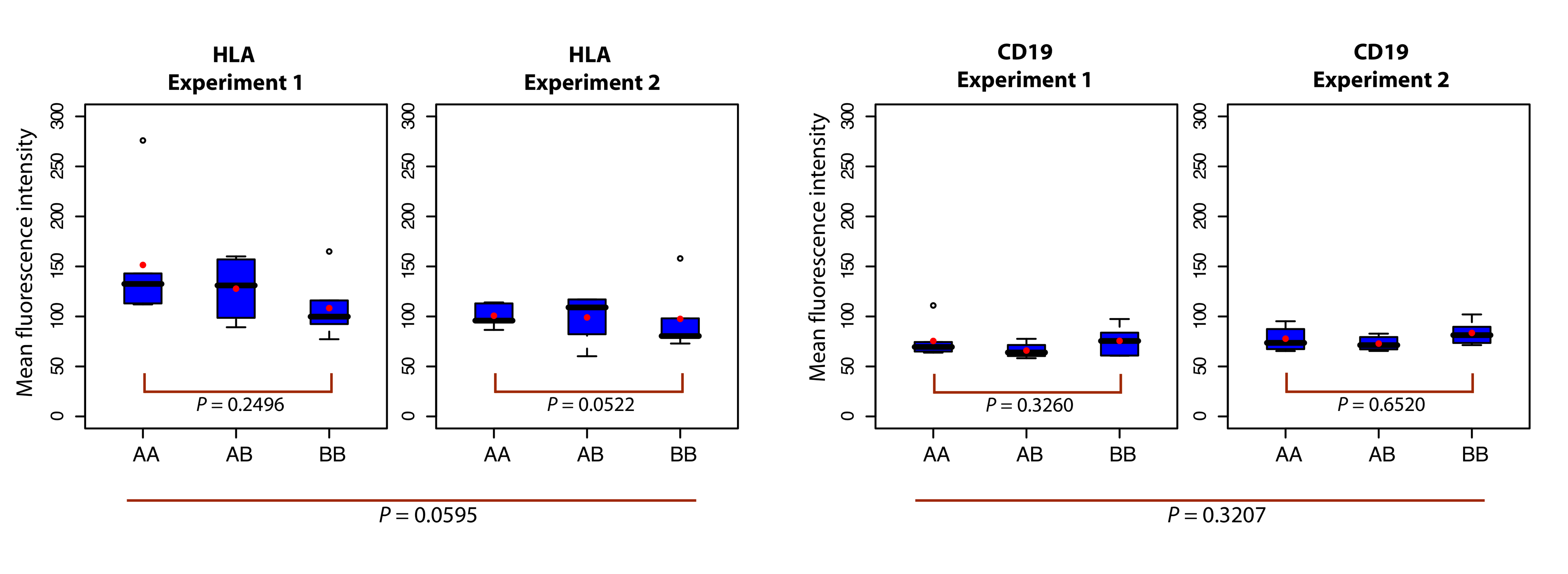

Supplement: Figure S8 — HLA-ABC and CD19 mean fluorescence intensities of B cells with various ERAP2 genotypes. The distribution of observed levels of surface-expressed HLA-ABC for B cells with AA, AB, and BB genotypes are graphically represented as boxplots (the blue box containing the 25th–75th percentile of the distribution, the black horizontal line indicating the median, the red dot reflecting the mean, and black circles representing outliers). HLA-ABC results are shown on the left, and CD19 results are shown on the right. Data are shown for two independent experiments (left and right in each case). For each experiment, the significance level of the comparison between AA and BB homozygotes (T-test) is shown within the plot; the significance level of the effect of genotype in the global comparison between AA and BB homozygotes (two-way ANOVA) is shown below. (0.27 MB TIF) [file pgen.1001157.s008.tif]

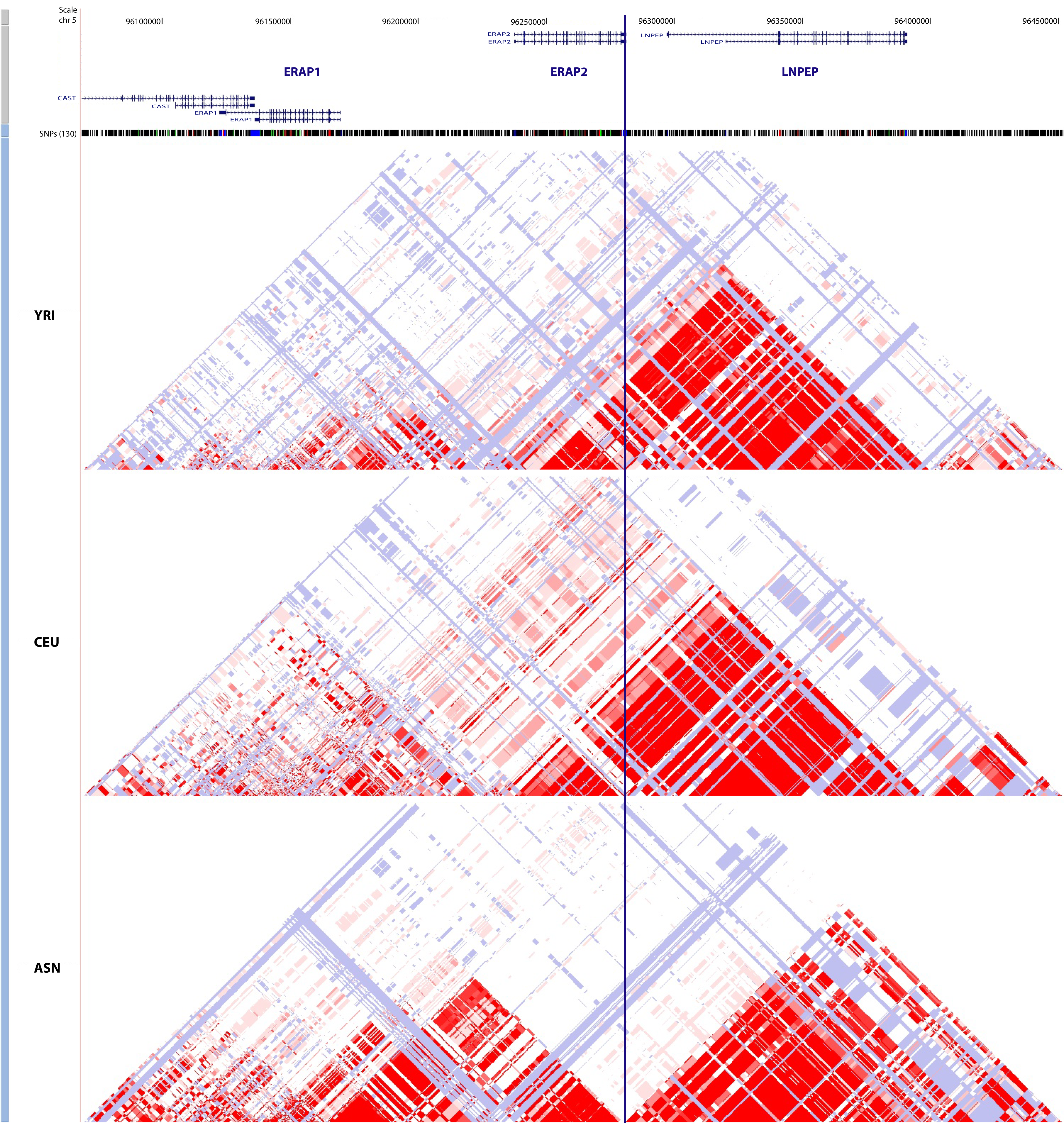

Supplement: Figure S9 — Linkage disequilibrium (LD) in the ERAP1, ERAP2, LNPEP genomic region based on HapMap polymorphism data. The strength of LD between a pair of SNPs is shown by the color of the diamond found at the intersection point connecting them: LD decreases from red to pink to blue to white (genome.ucsc.edu). YRI represents the Yoruba population, CEU the CEPH European sample, and ASN the Han Chinese and Japanese HapMap populations. (22.86 MB TIF) [file pgen.1001157.s009.tif]
