# Supplementary material for: Balancing Selection Maintains a Form of ERAP2 that Undergoes Nonsense-Mediated Decay and Affects Antigen Presentation
Source: PLoS Genet. 2010 Oct 14;6(10):e1001157. doi: 10.1371/journal.pgen.1001157 (PMC2954825; doi:10.1371/journal.pgen.1001157)
Supplement: Table S1 — Summary statistics and neutrality tests. S: number of SNPs; TajD: Tajima's D; p(TajD): P-value for Tajima's D test; p(MWU): P-value for MWUhigh test; FixedDiff: number of fixed differences with chimpanzee; p(HKA): P-value for HKA test. (0.10 MB DOC) [file pgen.1001157.s010.doc]

| **Dataset** | **Gene** | **Population** | **S** | **Theta** | **Pi** | **TajD** | **p(TajD)** | **p(MWU)** | **FixedDiff** | **p(HKA)** |
| --- | --- | --- | --- | --- | --- | --- | --- | --- | --- | --- |
| **All SNPs** | |  |  |  |  |  |  |  |  |  |
|  | ***ERAP1*** | |  |  |  |  |  |  |  |  |
|  |  | Yoruba | 52 | 11.30 | 11.92 | 0.19 | 0.032 | 0.048 |  |  |
|  |  | Luhya | 55 | 11.88 | 11.67 | -0.06 | 0.113 | 0.139 |  |  |
|  |  | Palestinian | 58 | 12.60 | 14.00 | 0.38 | 0.196 | 0.038 |  |  |
|  |  | Gujarati | 54 | 11.72 | 13.22 | 0.44 | 0.173 | 0.082 |  |  |
|  |  | Han | 41 | 8.82 | 11.23 | 0.91 | 0.027 | 0.010 |  |  |
|  |  | Toscani | 49 | 10.69 | 14.03 | 1.07 | 0.012 | 0.007 |  |  |
|  |  |  |  |  |  |  |  |  |  |  |
|  | ***ERAP2*** | |  |  |  |  |  |  |  |  |
|  |  | Yoruba | 45 | 9.78 | 15.68 | 2.05 | 0.000 | 0.000 |  |  |
|  |  | Luhya | 51 | 11.03 | 15.71 | 1.44 | 0.000 | 0.000 |  |  |
|  |  | Palestinian | 55 | 11.95 | 16.63 | 1.34 | 0.004 | 0.001 |  |  |
|  |  | Gujarati | 45 | 9.76 | 15.50 | 1.99 | 0.000 | 0.000 |  |  |
|  |  | Han | 38 | 8.22 | 14.83 | 2.68 | 0.000 | 0.000 |  |  |
|  |  | Toscani | 40 | 8.69 | 14.62 | 2.30 | 0.000 | 0.000 |  |  |
|  |  |  |  |  |  |  |  |  |  |  |
|  |  |  |  |  |  |  |  |  |  |  |
| **Coding SNPs** | |  |  |  |  |  |  |  |  |  |
|  | ***ERAP1*** | |  |  |  |  |  |  |  |  |
|  |  | Yoruba | 20 | 4.36 | 4.50 | 0.10 | 0.185 | 0.242 | 9 | 0.016 |
|  |  | Luhya | 19 | 4.10 | 4.31 | 0.16 | 0.158 | 0.201 | 9 | 0.023 |
|  |  | Palestinian | 22 | 4.78 | 4.89 | 0.08 | 0.435 | 0.311 | 8 | 0.000 |
|  |  | Gujarati | 22 | 4.78 | 5.05 | 0.18 | 0.382 | 0.327 | 9 | 0.000 |
|  |  | Han | 18 | 3.87 | 4.56 | 0.55 | 0.202 | 0.131 | 8 | 0.000 |
|  |  | Toscani | 17 | 3.71 | 5.10 | 1.16 | 0.057 | 0.037 | 8 | 0.002 |
|  |  |  |  |  |  |  |  |  |  |  |
|  | ***ERAP2*** | |  |  |  |  |  |  |  |  |
|  |  | Yoruba | 10 | 2.15 | 3.25 | 1.43 | 0.004 | 0.017 | 10 | 0.525 |
|  |  | Luhya | 11 | 2.37 | 3.16 | 0.95 | 0.026 | 0.145 | 10 | 0.400 |
|  |  | Palestinian | 13 | 2.81 | 3.82 | 1.05 | 0.094 | 0.028 | 10 | 0.019 |
|  |  | Gujarati | 12 | 2.57 | 3.51 | 1.05 | 0.105 | 0.068 | 10 | 0.033 |
|  |  | Han | 9 | 1.93 | 3.32 | 1.95 | 0.008 | 0.001 | 10 | 0.150 |
|  |  | Toscani | 11 | 2.36 | 3.34 | 1.17 | 0.078 | 0.085 | 10 | 0.067 |
