# Supplementary material for: Balancing Selection Maintains a Form of ERAP2 that Undergoes Nonsense-Mediated Decay and Affects Antigen Presentation
Source: PLoS Genet. 2010 Oct 14;6(10):e1001157. doi: 10.1371/journal.pgen.1001157 (PMC2954825; doi:10.1371/journal.pgen.1001157)
Supplement: Table S2 — dN/dS of ERAP2 and ERAP1. Estimated dN/dS ratios for the model that infers a single ratio for the whole phylogeny (Complete phylogeny) and estimated terminal branch dN/dS for the model that allows free ratios among branches (Lineage-specific). Dashes indicate species that lack the gene, while dots indicate species for which sequence could not be obtained. Likelihood ratio test results for the different analyses performed are in Table S3. (0.03 MB DOCX) [file pgen.1001157.s011.docx]

| **Lineage** | | ***ERAP2*** | ***ERAP1*** |
| --- | --- | --- | --- |
| **Complete phylogeny** | | 0.3258 | 0.1929 |
| **Lineage-specific** | |  |  |
|  | **Homo** | 0.335 | 0.164 |
|  | **Pan** | 0.107 | 0.100 |
|  | **Macaca** | 0.317 | . |
|  | **Callithrix** | 0.323 | 0.270 |
|  | **Mus** | -- | 0.209 |
|  | **Ratus** | -- | 0.260 |
|  | **Cavia** | -- | 0.164 |
|  | **Canis** | 0.384 | 0.257 |
|  | **Bos** | 0.260 | 0.224 |
|  | **Equus** | . | 0.140 |
