# Supplementary material for: Balancing Selection Maintains a Form of ERAP2 that Undergoes Nonsense-Mediated Decay and Affects Antigen Presentation
Source: PLoS Genet. 2010 Oct 14;6(10):e1001157. doi: 10.1371/journal.pgen.1001157 (PMC2954825; doi:10.1371/journal.pgen.1001157)
Supplement: Table S3 — Models of evolution used for analyzing ERAP2 and ERAP1. P-values of the log likelihood ratio test for all model comparisons performed (see Text S1). (0.04 MB DOC) [file pgen.1001157.s012.doc]

| **Model comparison** | **Model 1** | **Model 2** | **p(ERAP2)** | **p(ERAP1)** |
| --- | --- | --- | --- | --- |
|  |  |  |  |  |
| **Lineage heterogeneity** | one dN/dS | free dN/dS | 0.842 | 0.030 |
|  |  |  |  |  |
| **Purifying selection** | dN/dS = 1 | one dN/dS | 0.000 | 0.000 |
|  |  |  |  |  |
| **Primates different** | one dN/dS | primate dN/dS | 0.784 | 0.069 |
|  |  |  |  |  |
| **Human different** | one dN/dS | human dN/dS | 0.996 | 0.803 |
|  |  |  |  |  |
| **Site-specific positive selection** |  |  |  |  |
|  | model 1a | model 2 | 1 | 1 |
|  | model 7 | model 8 | 0.780 | 0.990 |
|  | model 8a | model 8 | 0.949 | 1 |
