# Supplementary material for: Balancing Selection Maintains a Form of ERAP2 that Undergoes Nonsense-Mediated Decay and Affects Antigen Presentation
Source: PLoS Genet. 2010 Oct 14;6(10):e1001157. doi: 10.1371/journal.pgen.1001157 (PMC2954825; doi:10.1371/journal.pgen.1001157)
Supplement: Table S4 — PCR primers. (0.04 MB DOC) [file pgen.1001157.s013.doc]

| **Experiment and orientation** | | **Primer** | **Location** |
| --- | --- | --- | --- |
|  | |  |  |
| cDNA amplification | |  |  |
|  | Forward | CCAACTCAAACAGGCAGATAAA | ERAP2 exon 1 |
|  | Reverse | GGGTTTATGCCATTTTCCTG | ERAP2 exon 19 |
|  | |  |  |
| Exon 10 splice variant | |  |  |
|  | Forward | CATTCGGATCCCAAGATGAC | ERAP2 exon 10 |
|  | Reverse | GGAGTGAACACCCGTCTTGT | ERAP2 exon 11 |
|  | |  |  |
| Genotyping | |  |  |
|  | Forward | gtgcacgttcagccaactaa | ERAP2 intron 9 |
|  | Reverse | aaagggacaggtgggctact | ERAP2 intron 10 |
