# Supplementary material for: Balancing Selection Maintains a Form of ERAP2 that Undergoes Nonsense-Mediated Decay and Affects Antigen Presentation
Source: PLoS Genet. 2010 Oct 14;6(10):e1001157. doi: 10.1371/journal.pgen.1001157 (PMC2954825; doi:10.1371/journal.pgen.1001157)
Supplement: Text S1 — Supporting materials. (0.11 MB DOC) [file pgen.1001157.s014.doc]

**Text S1. Supporting Materials Online**

**Balancing Selection Maintains a Form of *ERAP2* that Undergoes**

**Nonsense-Mediated Decay and Affects Antigen Presentation**

Aida M Andrés**^#^**, Megan Y Dennis**^#^**, , Jennifer L Cannons, Shih-Queen Lee-Lin, Belen Hurle, NISC Comparative Sequencing Program, Pamela L Schwartzberg, Scott H Williamson, Carlos D Bustamante,

Rasmus Nielsen, , and Eric D Green

**#** These authors contributed equally to this work.

**Control Regions**

The control regions in this study are unlinked (i.e., physically dispersed) known pseudogenes (Pseudogene.org) that are greater than 400 bp in length. Processed ribosomal pseudogenes and olfactory receptor pseudogenes were not included. To ensure that the selected regions are neutral with respect to evolutionary selection, each fulfills the following criteria: (1) does not overlap a known gene (among UCSC Genome Browser known genes); (2) is located over 2,000 bp from a known gene; (3) does not overlap evolutionarilly conserved regions (according to phastConsElements17way track on the UCSC Genome Browser); (4) is present as a pseudogene in chimpanzee, orangutan, and rhesus (according to Pseudogene.org and the reference genome sequence of each species [1,2, genome.ucsc.edu]; and (5) is present in a single copy in the human and chimpanzee genomes or, when a member of a gene family, only one member is included (only gene families with less than 90% sequence identity among members were considered, and only after manual verification of orthology was performed). The control regions do not include simple repeats or low complexity DNA, and they do not differ from the rest of the genome in terms of recombination rate (MWU *P-*value = 0.358 for Decode recombination rate; MWU *P-*value = 0.075 for Marshfield recombination rate; MWU *P-*value = 0.656 for Genethon recombination rate). Like genes, they have a higher GC content than non-coding regions (41% GC in genome, 47% GC in control regions, and 52% GC in coding genes).

**Absence of Signatures of a Recent Sweep in *ERAP2***

Very recent balancing selection resembles an incomplete sweep of positive selection: the (novel) selected allele increases in frequency rapidly, dragging along its unusually long and homogeneous haplotype. This signature of haplotype-specific long-range linkage disequilibrium (LD) is detected by the integrated haplotype score test, iHS [3]. We retrieved from the *Haplotter* web server (hg-wen.uchicago.edu/selection/haplotter.htm) the *ERAP2* iHS for HapMap Phase 2 populations (Yoruba, Han+Japanese, and CEPH [4]) and the graphical representation of *ERAP2* long-range haplotypes. For comparison, we also retrieved the iHS value of all HapMap Phase 2 SNPs. *ERAP2* does not show unusual long-range LD (Figure S3), and has typical iHS values in HapMap populations when compared with the rest of the genome: iHS (Yoruba) = 1.024, which corresponds to the 83rd percentile of the empirical genomic distribution of Yoruba iHS; iHS (Han+Japanese) = 1.377, which corresponds to the 43rd percentile of ASI iHS genomic distribution; and iHS (CEU) = -0.491, which corresponds to the 19th percentile of CEU iHS genomic distribution. Therefore, the gene does not show signatures of a recent incomplete sweep of positive or balancing selection.

**Conservation of *ERAP2* Haplotype Frequencies in Human Populations**

Our data suggest that 25% of the human population are AA homozygotes, 50% are AB heterozygotes, and 25% are BB homozygotes. Interestingly, these frequencies are rather consistent across the human groups present in the Human Genome Diversity Panel [5]: the haplotypes, as tagged by diagnostic SNP rs2549782, have a frequency between 0.4 and 0.6 in 47% of the 53 worldwide populations, and all populations have a frequency between 0.2 and 0.8 except for three [Papua (0.0294), Miao (0.1500), and Kalash (0.8913); www.genome.ucsc.edu].

**Comparative Genomics and PAML analysis**

We assessed the level of conservation of *ERAP1* and *ERAP2* across mammals by estimating their rate of protein evolution in the mammalian phylogeny. Briefly, we estimated *dN/dS*, where *dN* is the number of non-synonymous changes per non-synonymous site and *dS* is the number of synonymous changes per synonymous site. Because purifying selection affects primarily non-synonymous changes, *dN/dS* decreases with stronger purifying selection and increases with accelerated protein evolution.

Specifically, we obtained the coding sequence for the two genes from diverse mammals through queries of public databases, aligned the sequences, and manually reviewed the alignments to discard low-quality sequences and sequences without a clear open reading frame. This included obtaining the annotated *ERAP1* and *ERAP2* sequences from the UCSC Genome Browser [6], ENSEMBL [7], or RefSeq [8] databases for available mammalian species. For species that did not have an *ERAP1* or *ERAP2* gene annotated in these databases, we deduced the coding sequence of the gene using the human mRNA information and the orthologous genomic region of that species. For this, we used two different approaches. First, we used liftOver [9] to retrieve the genomic region orthologous to the human genomic region containing *ERAP1* or *ERAP2*, and mapped the human coding exons using Exonerate [10] to create the coding sequence (cds) orthologous to the human sequence. Second, we used liftOver to obtain the orthologous sequence to every human coding exon, and then reconstructed the complete orthologous cds. After aligning all sequences, we manually reviewed the alignment, and removed poorly aligning sequences, sequences with gaps due to missing data, and sequences without a good open reading frame. The final high-quality alignment contained sequences from *Homo sapiens*, *Pan troglodytes*, *Macaca mulatta*, *Callithrix jacchus*, *Canis familiaris*, *Bos taurus*, *Equus caballus*, *Mus musculus*, *Rattus norvegicus*, and *Cavia porcellus* for *ERAP1*, and *Homo sapiens*, *Pan troglodytes*, *Macaca mulatta*, *Callithrix jacchus*, *Canis familiaris*, and *Equus caballus* for *ERAP2* (Table S2). After taking into account the fact that *Mus musculus*, *Rattus norvegicus*, and *Cavia porcellus* genomes do not contain *ERAP2*, the number of sequences retrieved for *ERAP1* and *ERAP2* was very similar.

The two alignments were analyzed with *codeml* program in PAML 4 package [11]. We estimated the overall *dN/dS* for the complete tree, and compared the likelihood with models that allowed:

(1) free *dN/dS* for each branch;

(2) a primate-specific *dN/dS*; and

(3) a human-specific *dN/dS*.

In addition, we performed three tests aimed to detect site-specific signatures of positive selection across the phylogeny (branch models):

(1) comparison of model 2 (selection) vs. model 1a (neutral);

(2) model 8 (with *dN/dS* > 1) vs. model 7 (neutral); and

(3) model 8 (with *dN/dS* > 1) vs. model 8a (with *dN/dS* = 1).

Both *ERAP1* and *ERAP2* showed signatures of purifying selection, with low *dN/dS* (Table S2). Also, no gene showed evidence of differential constraint in primates or humans, or of site-specific positive selection (Tables S2 and S3).

**Identification of Alternative Splicing Variants in *ERAP2***

We performed an *in silico* analysis of the splicing forms of *ERAP2* to explore the correlation between *ERAP2* genotypes and mRNA splicing.

For this analysis, we used publicly available mRNA and EST data. We used MEGABLAST [12] to search the nr/nt database using as the query the coding sequence (cds) of the longest *ERAP2* reference sequence: bases 274-3156 of NCBI Reference Sequence NM_001130140.1. We only considered sequences that derived from mRNAs and unequivocally corresponded to human *ERAP2*. All sequences had a standardized bit score greater than 500 and a proportion of expected high-scoring segment pairs (E-value) smaller than 1e-99. We generated a multiple alignment of these sequences using MAFFT [13] and, by visual analysis, identified the following three insertions/deletions that corresponded to putative splicing variants:

1. ‘Variant 1’: a 135-bp deletion that completely spans exon 4 of the canonical *ERAP2* cds (detected in cDNA sequence BC065240.1);
2. ‘Variant 2’: a 56-bp insertion that derives from the 5’ extension of exon 10 (detected in cDNA sequences AY028805.1 and AB163917.1);
3. ‘Variant 3’: a 45-bp insertion that derives from the 5’ extension of exon 15 (detected in cDNA sequence AB163917.1).

To determine the level of support for each of these variants, we retrieved all available human ESTs derived from this region. Specifically, we performed a MEGABLAST search of the Human NCBI EST database using the sequence of each identified putative splicing variant (with 30 bp upstream and downstream of the exons involved) as the query. Apart from the aforementioned cDNAs, this search yielded one independent sequence (EST BG756499.1), which further supports Variant 2. This variant represents the extended exon 10 that we experimentally detected in the Haplotype B-derived mRNAs (see main text).

To establish whether Variant 2 was consistently associated with one major *ERAP2* haplotype, we determined the allele in each sequence for the 4 coding diagnostic SNPs. The two cDNA sequences that contained Variant 2 differed from the human reference sequence at all coding diagnostic SNPs. Specifically, they agreed with the Haplotype B sequence. EST BG756499.1, which contains the extended exon 10, also agreed with the Haplotype B sequence.

**Predicting the Effect of rs2248374 in *ERAP2* mRNA Splicing**

To better understand the role of rs2248374 and other *ERAP2* sequence variants in mRNA splicing, we used a gene-prediction method to predict the splicing of *ERAP2* mRNAs (GeneID [14]). Analysis of Haplotype A correctly predicted the standard exon 10. Analysis of Haplotype B yielded a prediction that skips exon 10 due to the strict limitations of the method with respect to protein-coding potential (GeneID considers coding potential information, and it cannot predict an extended exon 10 that contains two STOP codons). When the two STOP codons were removed from the sequence, GeneID correctly predicted the extended exon 10 on the Haplotype B sequence. Moreover, when the rs2248374-G allele was introduced in an otherwise Haplotype A background, GeneID predicted the extended exon 10, showing that this SNP can itself explain the difference in splicing for mRNAs derived from the two haplotypes.

**Functional Linkage of *ERAP2* with *ERAP1* and *LNPEP***

We aimed to identify putatively functional variants in *LNPEP* linked to *ERAP2* common polymorphisms, but could not detect any *LNPEP* functional difference that associated with *ERAP2* Haplotype A or B. Specifically, no *LNPEP* coding variant was found in high LD with *ERAP2* diagnostic SNPs; further, we determined that the two known common *LNPEP* mRNA isoforms (GenBank Accessions U62768 and U62769) are not associated with the *ERAP2* Haplotype A or B (as shown by PCR analysis of *LNPEP* cDNA prepared from LCLs of known *ERAP2* genotype).

Likewise*,* we found no apparent *ERAP1* functional variants linked to *ERAP2* Haplotype A or B. First, no *ERAP1* coding SNP is in high LD with *ERAP2* diagnostic SNPs. Second, we could not identify differences in the amounts or the size of ERAP1 protein (as shown by western blot analysis of LCL lysates with known *ERAP2* genotype) that correlated with *ERAP2* haplotypes, demonstrating that *ERAP1* expression and splicing are independent of *ERAP2* variants. It is worth noting that we could not perform allele-specific gene expression analyses of *LNPEP* or *ERAP1* since none of these genes’ coding SNPs are in high LD with *ERAP2* diagnostic SNPs*.*

**ERAP2 in Immortalized Cell Lines**

Different studies have yielded contradictory results regarding the presence of ERAP2 protein in human cell lines. For example, some groups detected ERAP2 protein in HeLa cells, while others did not (reviewed in [15]). We analyzed HeLa cells and found this cell line to be a BB-homozygote and to produce the alternatively spliced NMD transcript for *ERAP2* (data not shown). We also find very limited expression of the full-length ERAP2 protein in HeLa protein extracts (Figure S4). These conflicting observations may be due to clonal differences among HeLa cell lines, which could have different *ERAP2* genotype, or different mechanisms for splicing and NMD. Further studies are required to clarify this issue.

Additionally, previous studies detected truncated ERAP2 protein when the *ERAP2* cDNA was transfected into cells [16], likely because the transfected mRNA saturated the NMD capacity of the cell. Nevertheless, Fruci et al. [17] performed western-blot analysis of tumors and detected a band that might correspond to the truncated ERAP2 protein. Although the gel was not shown in this paper, and a direct comparison with our results is not possible, perhaps transformed cells have aberrant splicing and/or NMD processes that affect the processing of Haplotype B-derived *ERAP2* mRNA.

In this regard, *ERAP2* expression in tumors is not always well-correlated with levels of ERAP2 protein or with MHC class I expression [17-19]. While altered splicing and/or NMD mechanisms in tumors or immortalized cells may contribute to these observations, we propose that *ERAP2* genotype differences among the cells analyzed may account for some of these findings.

**References**

1. Consortium CSaA (2005) Initial sequence of the chimpanzee genome and comparison with the human genome. Nature 437: 69-87.

2. Gibbs RA, Rogers J, Katze MG, Bumgarner R, Weinstock GM, et al. (2007) Evolutionary and biomedical insights from the rhesus macaque genome. Science 316: 222-234.

3. Voight BF, Kudaravalli S, Wen X, Pritchard JK (2006) A map of recent positive selection in the human genome. PLoS Biol 4: e72.

4. Consortium TIH (2003) The International HapMap Project. Nature 426: 789-796.

5. Cann HM, de Toma C, Cazes L, Legrand MF, Morel V, et al. (2002) A human genome diversity cell line panel. Science 296: 261-262.

6. Rhead B, Karolchik D, Kuhn RM, Hinrichs AS, Zweig AS, et al. (2010) The UCSC Genome Browser database: update 2010. Nucleic Acids Res 38: D613-619.

7. Hubbard TJ, Aken BL, Ayling S, Ballester B, Beal K, et al. (2009) Ensembl 2009. Nucleic Acids Res 37: D690-697.

8. Pruitt KD, Tatusova T, Maglott DR (2007) NCBI reference sequences (RefSeq): a curated non-redundant sequence database of genomes, transcripts and proteins. Nucleic Acids Res 35: D61-65.

9. Kent WJ, Sugnet CW, Furey TS, Roskin KM, Pringle TH, et al. (2002) The human genome browser at UCSC. Genome Res 12: 996-1006.

10. Slater GS, Birney E (2005) Automated generation of heuristics for biological sequence comparison. BMC Bioinformatics 6: 31.

11. Yang Z (2007) PAML 4: phylogenetic analysis by maximum likelihood. Mol Biol Evol 24: 1586-1591.

12. Zhang Z, Schwartz S, Wagner L, Miller W (2000) A greedy algorithm for aligning DNA sequences. J Comput Biol 7: 203-214.

13. Katoh K, Asimenos G, Toh H (2009) Multiple alignment of DNA sequences with MAFFT. Methods Mol Biol 537: 39-64.

14. Parra G, Blanco E, Guigo R (2000) GeneID in Drosophila. Genome Res 10: 511-515.

15. Saveanu L, Carroll O, Hassainya Y, van Endert P (2005) Complexity, contradictions, and conundrums: studying post-proteasomal proteolysis in HLA class I antigen presentation. Immunol Rev 207: 42-59.

16. Tanioka T, Hattori A, Masuda S, Nomura Y, Nakayama H, et al. (2003) Human leukocyte-derived arginine aminopeptidase. The third member of the oxytocinase subfamily of aminopeptidases. J Biol Chem 278: 32275-32283.

17. Fruci D, Ferracuti S, Limongi MZ, Cunsolo V, Giorda E, et al. (2006) Expression of endoplasmic reticulum aminopeptidases in EBV-B cell lines from healthy donors and in leukemia/lymphoma, carcinoma, and melanoma cell lines. J Immunol 176: 4869-4879.

18. Fruci D, Giacomini P, Nicotra MR, Forloni M, Fraioli R, et al. (2008) Altered expression of endoplasmic reticulum aminopeptidases ERAP1 and ERAP2 in transformed non-lymphoid human tissues. J Cell Physiol 216: 742-749.

19. Kamphausen E, Kellert C, Abbas T, Akkad N, Tenzer S, et al. (2010) Distinct molecular mechanisms leading to deficient expression of ER-resident aminopeptidases in melanoma. Cancer Immunol Immunother 59: 1273-1284.
